# Population diversity of the genetically determined *TTR* expression in human tissues and its implications in TTR amyloidosis

**DOI:** 10.1186/s12864-017-3646-1

**Published:** 2017-03-23

**Authors:** Andrea Iorio, Flavio De Angelis, Marco Di Girolamo, Marco Luigetti, Luca G. Pradotto, Anna Mazzeo, Sabrina Frusconi, Filomena My, Dario Manfellotto, Maria Fuciarelli, Renato Polimanti

**Affiliations:** 1 Department of Biology, University of Rome Tor Vergata, Rome, Italy; 2 Clinical Pathophysiology Center, AFaR Foundation – “San Giovanni Calibita” Fatebenefratelli Hospital, Isola Tiberina, Rome, Italy; 3 Departments of Geriatrics, Neurosciences & Orthopedics, Institute of Neurology, Catholic University of the Sacred Heart, Fondazione Policlinico Universitario A. Gemelli, Rome, Italy; 4 Division of Neurology and Neurorehabilitation, San Giuseppe Hospital, IRCCS-Istituto Auxologico Italiano, Piancavallo (VB), Italy; 5 Department of Clinical and Experimental Medicine, University of Messina, Messina, Italy; 6 Genetic Diagnostics Unit, Laboratory Department, Careggi University Hospital, Florence, Italy; 7 Division of Neurology, “Vito Fazzi” Hospital, Lecce, Italy; 8 Department of Psychiatry, Yale University School of Medicine and VA CT Healthcare Center, VA CT 116A2, 950 Campbell Avenue, West Haven, CT 06516 USA

**Keywords:** Transthyretin, Genotype-phenotype correlation, Mutation, Amyloid, Gene expression

## Abstract

**Background:**

Transthyretin (TTR) amyloidosis is a hereditary disease with a complex genotype-phenotype correlation. We conducted a literature survey to define the clinical landscape of TTR amyloidosis across populations worldwide. Then, we investigated whether the genetically determined *TTR* expression differs among human populations, contributing to the differences observed in patients. Polygenic scores for genetically determined *TTR* expression in 14 clinically relevant tissues were constructed using data from the GTEx (Genotype-Tissue Expression) project and tested in the samples from the 1,000 Genomes Project.

**Results:**

We observed differences among the ancestral groups and, to a lesser extent, among the investigated populations within the ancestry groups. Scandinavian populations differed in their genetically determined *TTR* expression of skeletal muscle tissue with respect to Southern Europeans (*p* = 6.79*10^−6^). This is in line with epidemiological data related to Swedish and Portuguese *TTR* Val30Met endemic areas. Familial amyloidotic cardiomyopathy (TTR deposits occur primarily in heart tissues) presents clinical variability among human populations, a finding that agrees with the among-ancestry diversity of genetically determined *TTR* expression in heart tissues (i.e., Atrial Appendage *p* = 4.55*10^−28^; Left Ventricle *p* = 6.54*10^−35^).

**Conclusions:**

Genetically determined *TTR* expression varied across human populations. This might contribute to the genotype-phenotype correlation of TTR amyloidosis.

**Electronic supplementary material:**

The online version of this article (doi:10.1186/s12864-017-3646-1) contains supplementary material, which is available to authorized users.

## Background

Transthyretin (TTR) amyloidosis (OMIM: 105210) is a rare, life-threatening, progressively debilitating, autosomal dominant condition characterized by extracellular deposition of TTR-derived amyloid fibrils in peripheral and autonomic nervous system, heart, and other organs, leading to tissue damage and organ failure [1, 2]. The disorder is caused by point mutations in the *TTR* gene (NM_000371) located in chromosome region 18q12.1 [3]. The disease presents multiple clinical signs, including peripheral neuropathy (sensory and motor), autonomic neuropathy, gastrointestinal impairment, cardiomyopathy, nephropathy, and ocular deposition [4]. While these symptoms may be present in patients with different *TTR* mutations, phenotypes are not always concordant and the same point mutation may be associated with different signs/symptoms [5]. The clinical heterogeneity of the carriers of the *TTR* amyloidogenic mutations is particularly relevant from a population perspective. The most striking example of this inter-population diversity is the Val30Met mutation [rs28933979, c.148G > A, p.Val50Met], which is one of the leading causes of TTR amyloidosis [6, 7]. In the two European Val30Met endemic areas (i.e., Portugal and Sweden), Val30Met patients show two distinct phenotypic presentations. In Val30Met Portuguese families, the disease shows early-onset, strong severity, and high penetrance [8, 9], whereas Val30Met Swedish patients have late-onset, intermediate severity, and low penetrance [10]. This complex genotype-phenotype correlation indicates that the clinical presentation is not only regulated by the disease-causing mutation. The amyloidogenic mutation is the cause of the disease, but other factors contribute to the modulation of the disease phenotype [11–13]. Our previous investigations focused on the role of non-coding variation in the genotype-phenotype correlation of TTR amyloidosis. We observed an enrichment for non-coding regulatory variants located in heart-related transcription-factor binding sites in African populations, suggesting a contribution to the cardiomyopathy observed in patients of African ancestry [12]. We also investigated the haplotype structures of Val30Met and Val122Ile [rs76992529, c.424G > A, p.Val142Ile] mutations, observing in both cases independent haplotypes carrying the same disease-causing mutation [14, 15]. Non-coding variation regulates genome functions, especially through its key role in transcriptional mechanisms across human tissues [16]. On this basis, we hypothesized that, in presence of an amyloidogenic mutation, the non-coding regulation of *TTR* gene expression across tissues contributes the distribution of TTR-derived amyloid fibrils and, consequently, the disease presentation. In accordance with this hypothesis, a recent study demonstrated that, although the liver is the main TTR organ source, *TTR* gene expression in other tissues can also be involved in the processes related to the disease phenotype [17].

Recent studies have focused on how gene expression regulates relevant biological processes and how its alteration can lead to the onset of diseases [18]. The Genotype-Tissue Expression (GTEx) Project is investigating genetic variation in relation to gene expression in human tissues [19]. GTEx data (available at *http://www.gtexportal.org/*) provide information about the relationship between genetic variations and gene expression in 43 different human tissues [20]. The effects of genetic variants can be used to estimate the genetically determined gene expression to investigate the role of gene expression in multiple tissues with respect to disease pathogenesis [21, 22]. Accordingly, the analysis of the genetically determined *TTR* expression can help to discern its involvement in human tissues with respect to the genotype-phenotype correlation of TTR amyloidosis.

## Results

### Results from literature review

We identified 88 worldwide disease-causing mutations with information regarding the ancestry (Additional file 1). Our findings indicated that Europeans have the highest number of *TTR* mutations (*N* = 60), followed by East Asians (*N* = 27), Americans (*N* = 20), Central-South Asians (*N* = 8) and Africans (*N* = 3). The ancestry was not specified for the remaining amyloidogenic mutations. Few mutations are reported in multiple ancestry groups (e.g., Val30Met and Val122Ile) and several symptoms are reported for patients with different ancestries and different mutations (e.g., cardiomyopathy and sensorimotor neuropathy) (Additional file 1). However, clinical signs partially occur in an ancestry-specific manner with respect to the amyloidogenic mutation reported (Additional file 1).

### Genetically-predicted TTR expression

As introduced above, we used the data from GTEx Project [19] to build polygenic scores associated with *TTR* expression in 14 human tissues and tested them in the samples from the 1,000 Genomes Project [23] considering both among-ancestry and within-ancestry analyses. A detailed description of procedures used is reported in the method section.

### Among-ancestry comparisons

The among-ancestry comparisons showed very significant differences (*p* < 2.89*10^−9^) for genetically predicted *TTR* expression scores for all investigated tissue (Additional file 2) with the exception of the Esophagus – Muscularis tissue (*p* > 0.05). Post-hoc pairwise analysis of the among-ancestry comparisons indicated that these significant differences are generally present across multiple ancestries and are not due to the diversity of a single population (Additional file 3). The only exception to this general trend is the Colon - Transverse tissue where the significant result is exclusively driven by the difference between African and non-African populations (*p* = 5.44*10^−11^).

### Within-ancestry comparisons

**Fig. 1:**
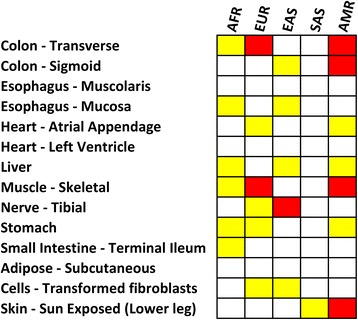
Heatmap of the Kruscal-Wallis results related to the within-ancestry comparisons. The colors refer to different significance levels (*red*: Bonferroni-corrected significance; *yellow*: Nominal significance). (AFR: Africa, EUR: Europe, EAS: East Asia, SAS: South Asia, AMR: America)

**Fig. 2:**
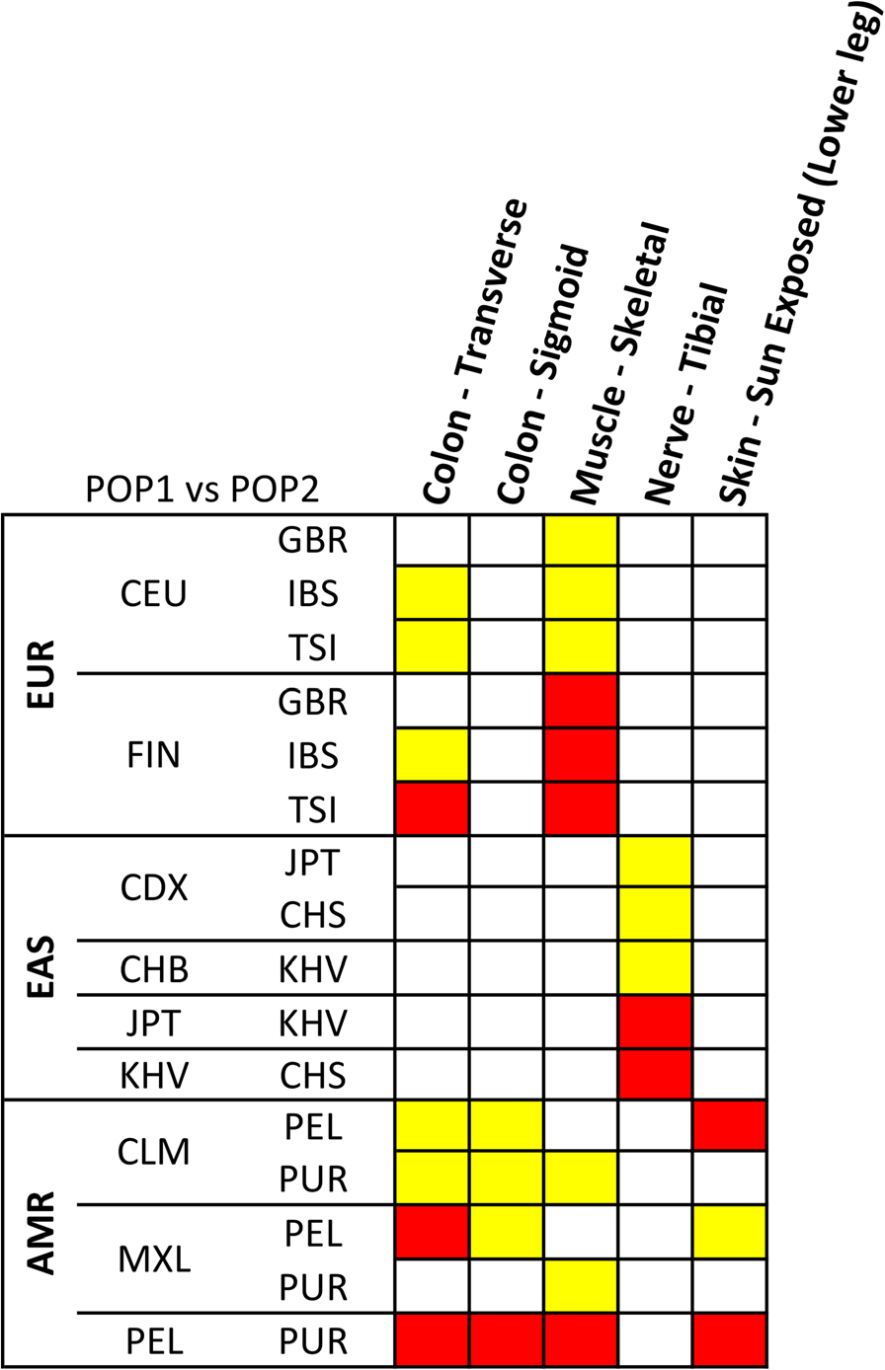
Heatmap of the Kruscal-Wallis post-hoc analysis results of within-ancestry comparisons. The colors refer to different significance levels (*red*: Bonferroni-corrected significance; *yellow*: Nominal significance). Information about population definitions are available at http://www.1000genomes.org/about (EUR: Europe, EAS: East Asia, AMR: America)

**Fig. 3:**
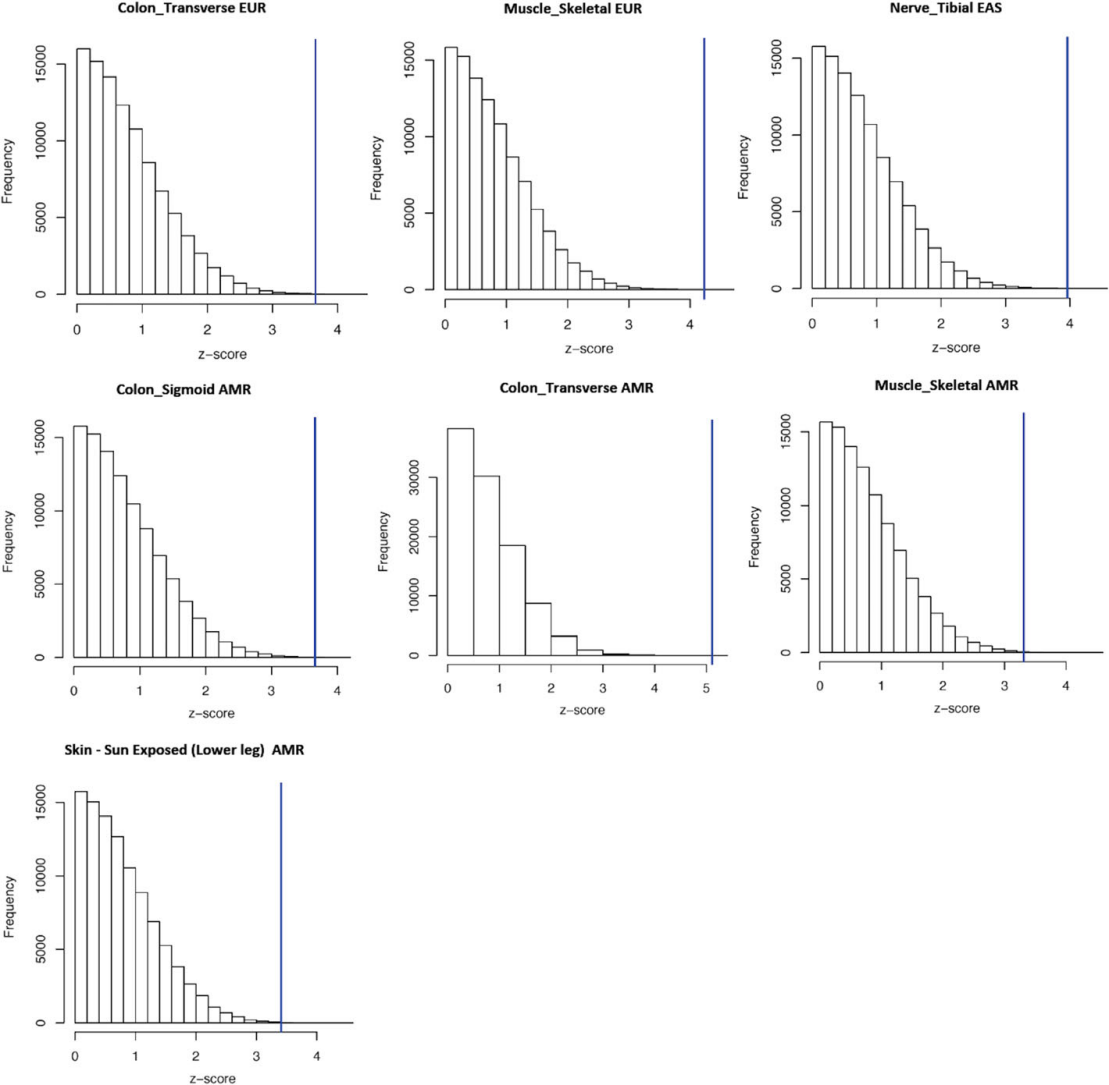
Distribution of the z-scores generated from 100,000 random permutations with respect to the z-scores observed in the Kruscal-Wallis post-hoc analysis of within-ancestry comparisons. (EUR: Europe, EAS: East Asia, AMR: America)

Within-ancestry comparisons showed less tissue- and ancestry-specific differences than among-ancestry comparisons (Fig. 1). Significant differences were observed within European ancestry (Colon – Transverse *p* = 0.002 and Muscle – Skeletal *p* = 6.79*10^−6^), within Eastern Asian ancestry (Nerve - Tibial *p* = 7*10^−5^), and within American ancestry (Colon - Transverse *p* = 3.2*10^−7^, Colon – Sigmoid *p* = 2*10^−4^, Muscle – Skeletal *p* = 9*10^−4^, and Skin - Sun Exposed (Lower leg) (*p* = 6*10^−4^). The significant results of the post-doc pairwise analysis are reported in Fig. 2. In European populations, the diversity of *TTR* expression scores in the significant tissues is driven by North–south variability, with the most significant diversity between Scandinavian populations (i.e., the 1,000 Genomes Project FIN population) and the other European samples (Additional file 4). In Eastern Asian samples, the diversity for *TTR* expression scores in Nerve - Tibial tissue is driven by differences of Vietnamese populations (i.e., the 1,000 Genomes Project KHV population) with respect to Japanese and Chinese populations (Additional file 5). Regarding the tissues identified in the American samples, the diversity of TTR expression scores is driven by differences between Peruvian population (i.e., the 1,000 Genomes Project PEL population) and other American populations (Additional file 6). Permutation analysis confirmed that all within-ancestry observed differences significantly diverge from the null distribution of the permuted results (Fig. 3). The observed Z-scores are located in extremely marginal positions with respect to the null distribution of the Z-scores generated by the random permutations.

No significant differences were observed for African ancestry (nominal significance for Colon –Transverse, Esophagus – Muscularis, Liver, Muscle – Skeletal, Stomach, and Small Intestine; Additional file 7) and Central-South Asian ancestry (nominal significance for the Skin - Sun Exposed (Lower leg) tissue; Additional file 8).

## Discussion

**Fig. 4:**
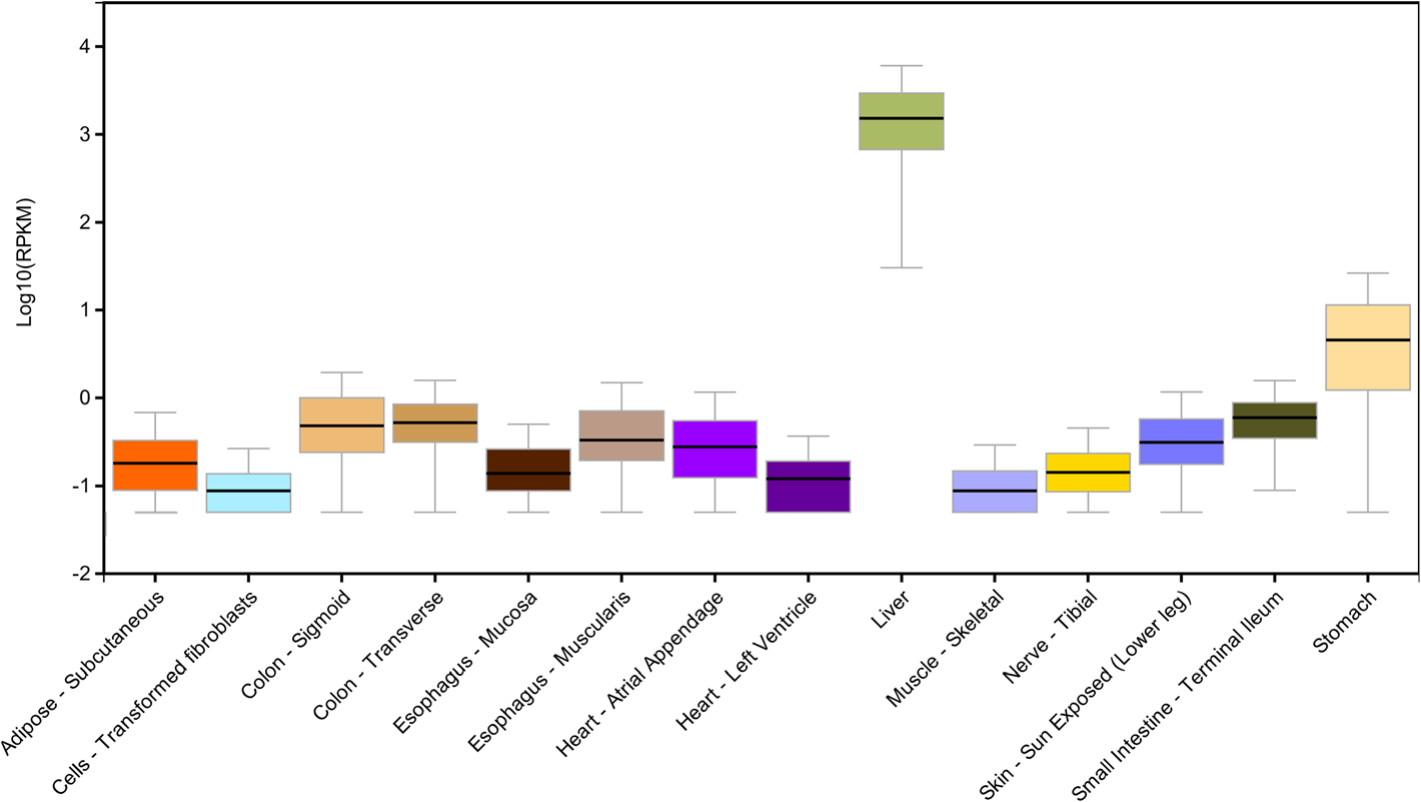
*TTR* gene expression across the 14 clinically relevant tissues investigated the present study. This figure was extracted from the GTEx portal available at http://www.gtexportal.org/home/

Our literature review indicated that few mutations were observed in multiple ancestral groups (e.g., Val30Met and Val122Ile). However, these are the mutations detected in most patients affected by TTR amyloidosis and the corresponding clinical signs mainly occur with ancestry-specific patterns. Although these ancestry differences are likely biased by the rare disease prevalence and the variability of the clinical practice guidelines across different countries, the inter-population diversity of the molecular mechanisms involved in the genotype-phenotype correlation surely plays an important role in the clinical presentation observed in patients with different ancestry backgrounds. Our previous investigations indicated that *TTR* non-coding regions are affected by human population diversity with potential consequences on gene regulation [12, 14, 15]. Our hypothesis is in agreement with many studies about the regulatory role of non-coding variation on gene expression and other gene functions [24, 25]. *TTR* gene expression showed a relevant inter-individual variability across human tissues (Fig. 4), and the related tissue-specific regulatory mechanisms is likely to be one of the processes involved in the disease genotype-phenotype correlation. Our current findings based on a large multi-ethnic cohort (*N* = 2,504) and gene expression information from multiple human tissues (*N* = 14) provide novel insight regarding the regulatory mechanisms of *TTR* gene. Indeed, very few investigations explored mechanisms related to gene expression in TTR amyloidosis due to limited availability of tissue samples from affected patients. In 2014, Norgren and colleagues [26] observed that *TTR* gene expression is significantly higher in patients’ liver than in healthy controls. They hypothesized an impaired endoplasmatic reticulum-associated degradation and posited that the endoplasmatic reticulum-assisted folding was caused by an overload of mutated TTR protein. Recently, an in vitro study demonstrated that Schwann cells can contribute to neurodegeneration in TTR amyloidosis through the local expression of mutated TTR [17]. Accordingly, *TTR* gene expression patterns across different tissues, including source and target organs, can contribute to the symptoms observed in patients. To provide novel information regarding this topic, we used data from the GTEx project and the 1,000 Genomes Project. Specifically, we calculated tissue-specific scores to link genetic variability to *TTR* gene expression and analyzed the inter-population variability considering both differences among ancestries and among populations within the same ancestral groups. Our data confirmed that non-coding variations affect gene expression with tissue-specific patterns and that human populations have significant differences. Due to the very low disease prevalence and relatively few reports regarding TTR amyloidosis, it is difficult to conduct effective comparisons between epidemiological and molecular data. However, in some cases, we observed consistency between clinical evidences and our computational results.

Since Val30Met is the most recurring mutation in patients with TTR amyloidosis, numerous epidemiological studies investigated its distribution across human populations. As mentioned above, the features of the two endemic Val30Met foci in Europe are well known: Swedish patients with late-onset, intermediate severity, and low penetrance vs. Portuguese patients with early-onset, severe symptoms, and high penetrance [7]. Our molecular outcomes are consistent with these epidemiological data: Scandinavian populations (i.e., the 1,000 Genomes Project FIN) showed the strongest difference with respect to southern European populations (i.e., the 1,000 Genomes Project IBS and TSI) for Muscle – Skeletal tissue.

The second most recurrent *TTR* mutation is Val122Ile that reaches 4% in African-Americans and West Africans [27]. This mutation is mainly associated with familial amyloidotic cardiomyopathy due to TTR deposits in heart tissue [28]. In our literature survey, we observed that Val122Ile was reported in multiple ancestry groups with high heterogeneity in the disease features related to the familial amyloidotic cardiomyopathy (e.g., onset and severity). Regarding heart tissues (i.e., Heart - Atrial Appendage; Heart - Left Ventricle), we observed significant differences among the ancestry groups investigated that may be in agreement with the epidemiological data collected.

Besides this consistent overlapping of our results with known epidemiological evidences, we observed other strong differences within some ancestry groups that may support future epidemiological investigations. In the East Asian group, our analysis revealed a significant difference between Vietnamese population (i.e., the 1,000 Genomes Project KHV population) and Japanese and Chinese groups (i.e., the 1,000 Genomes Project JPT and CHS populations) that agrees with previous studies on the genetic structure of East Asian populations [29]. Regarding TTR amyloidosis, Japan is one of the endemic foci of the disease with a prevalence of one per million, and different mutations have been identified along with a marked heterogeneity in the disease phenotypic expression [30]. Chinese cases with different TTR mutations (e.g., Gly83Arg, Ile107Met) have also been identified [31]. To our knowledge, no reports have been published on Vietnamese or other South-Eastern Asian populations. Further studies on Eastern Asian patients may indicate strong differences within this ancestry group in accordance with our data. Another intriguing result is related to the admixed American populations of the 1,000 Genomes Project (i.e., CLM, MXL, PEL, PUR). These population clusters are an admixture of European, African, and Native American ancestry and a recent study indicated strong differences in the admixture proportions [32]. Previous studies demonstrated that haplotype structure of admixed populations play an important role in gene regulatory mechanisms [33, 34]. Our current data suggested that admixture differences could contribute to the heterogeneity observed among patients from admixed American populations.

Beyond TTR hereditary amyloidosis, non-coding variants associated with *TTR* expression could be involved in the pathogenesis of the non-inherited form of TTR amyloidosis, known as senile systemic amyloidosis. This disorder is caused by a deposition of fibrils derived from TTR in subjects that do not carry amyloidogenic mutations. It occurs as cardiomyopathy in elderly men with European ancestry, and TTR amyloid fibrils can be found in the hearts of the 25% of elderly individuals over 80 years of age [35]. A recent study provided suggestive evidences regarding the role of non-coding regulatory regions in wild-type TTR amyloidosis [36]. Together with these previous findings, our data suggest that *TTR* non-coding variation and its effect on transcription regulation are strong candidates as casual factors in the non-inherited form of TTR amyloidosis.

## Conclusions

In conclusion, the current study advances the knowledge of TTR amyloidosis in terms of both data regarding the inter-population variability of the disease and methodology that can be applied. However, our results are affected by some limitations. The GTEx Project investigated a multi-ethnic cohort with limited sample size that cannot completely detect the effects of genetic variability on gene expression across human populations. Although our findings provided insights regarding TTR expression regulation, our analysis is based on data from general-population cohort that included subjects without *TTR* mutations. Therefore, our findings do not account for interactions between the amyloidogenic mutation and *TTR* gene regulation, which likely contribute to TTR expression variability in the affected patients. Due to the large sample size (*N* = 2,504) used to investigate the role of non-coding variation in the regulation of TTR expression across human tissue, the possibility to experimentally confirm our findings is currently limited by cost and sample availability. Finally, in addition to *TTR* gene expression, other mechanisms can also contribute to the genotype-phenotype correlation of the disease, and our data may only reflect one of the molecular processes involved. Further in vivo and in vitro investigations are warranted to follow up our results and confirm the role of genetically determined *TTR* expression in the disease onset and progression.

## Methods

### Literature survey

To delineate the genetic and clinical landscape of this disease among worldwide populations, we used PubMed to identify 938 scientific articles related to TTR amyloidosis. The literature search was performed in January 2016 with the following key words: “TTR”, “TTR amyloidosis”, “TTR mutation”, “TTR gene”. Of these 938 papers, we selected studies (*n* = 144) with information concerning clinical signs and *TTR* mutations of patients investigated. Finally, we partitioned the selected articles by ancestral group: Africa (Africans and African-Americans patients), Europe, Central-South Asia, East Asia, and America. In the American group, we included those studies involving patients of Hispanic ethnicity and/or Native American ancestry. In accordance with the vast majority of the literature regarding TTR amyloidosis, we named each TTR mutation in accordance with the protein change in the mature protein. We also reported rsID (when available) and the protein change in the protein precursor (Additional file 1).

### Genotype and expression data

Phase 3 of the 1,000 Genomes Project was considered the reference genotype dataset [23]. We obtained the VCF (Variant Call Format) file of the 40 Kb region, which includes upstream region, *TTR* CDS, and downstream region (GRCh37/hg19 chr 18: 29155000–29195000). Detailed information about population definitions is available at *http://www.1000genomes.org/about*. The VCF file of the investigated region can be downloaded from the following link: http://phase3browser.1000genomes.org/Homo_sapiens/Location/View?r=18%3A29155000-29195000.

The GTEx Version 6 data were used as reference datasets for genetically determined gene expression [20]. GTEx cohort includes individuals with different ancestry and it was previously used for population comparisons [37]. We extracted information regarding the effects (i.e., beta values and p-values) of genetic variants on *TTR* gene expression in 14 clinically relevant tissues among those available in GTEx data: Colon – Transverse; Colon – Sigmoid; Esophagus –Muscularis; Esophagus – Mucosa; Heart - Atrial Appendage; Heart - Left Ventricle; Liver; Muscle – Skeletal; Nerve – Tibial; Stomach; Small Intestine - Terminal Ileum; Adipose – Subcutaneous; Cells - Transformed fibroblasts; and Skin - Sun Exposed (Lower leg). Finally, we identified 132 variants (131 non-coding variants e 1 coding variant) presenting comprehensive information for the 14 tissues. In the Additional file 9, we reported the GTEx statistics used to build the tissue-specific polygenic scores. The original GTEx data used in the current study can be obtained from the following link: http://www.gtexportal.org/home/testyourown.

### Data analysis

The first step of the analysis was to build polygenic scores for genetically determined *TTR* expression for each of the 14 clinically relevant tissues. These tissue-specific polygenic scores were a sum of alleles associated with *TTR* expression in a specific tissue, weighted by effect sizes. As mentioned above, we used 1,000 Genomes Project data as reference dataset for LD (Linkage Disequilibrium) structure and human genetic variability and GTEx data as reference datasets to determine the effect of genetic variants upon *TTR* expression. We conducted a LD clumping analysis using Plink 1.07 toolset [38]. We included in the analysis SNPs (Single Nucleotide Polymorphisms) with at least a trend effect (*p* ≤ 0.1) on *TTR* expression and considered standard LD parameters (r^2^ = 0.5, and region size = 10Kb). The LD clumping was conducted with respect to two perspectives: comparisons among ancestry groups (i.e., among-ancestry comparisons) and comparisons across population within ancestry groups (i.e., within-ancestry comparison). Accordingly, we calculated 14 tissue-specific clumped datasets for each ancestry (i.e., LD information across all ancestry) and 14 tissue-specific clumped datasets for the populations within each ancestry groups (i.e., LD information specific for each ancestry group). All calculated datasets consisted of genetic variants in non-coding regions of *TTR* gene and the composition of each dataset is reported in the Additional file 10. We used these tissue-specific clumped datasets to calculate the polygenic scores for genetically determined *TTR* expression on the basis of effect-allele count and allele effect size.

The among-ancestry and within-ancestry comparisons were performed using the Kruskal-Wallis test. This non-parametric test permitted us to verify whether the differences observed among ancestries and among populations within the same ancestries were statistically significant. To deepen the findings obtained from the Kruskal-Wallis analysis, we used Dunn’s test for the post-hoc pairwise comparisons. Bonferroni correction accounting for the number of tissues tested was applied to adjust the results for multiple-testing comparisons. Finally, we further quantified the significance of the observed within-ancestry differences, conducting a permutation analysis. Specifically, we performed 100,000 permutations of the individual tissue-specific polygenic scores with respect to their population origins and tested whether the observed differences were significantly different from the null distribution of the permuted results.

## Additional files

Additional file 1: Inter-ethnic clinical diversity associated with TTR mutations. Each mutation is named in accordance of the missense substitution in the mature protein. For each mutation, rsID and protein change in the protein precursor is also reported. (PDF 137 kb)

Additional file 2: Kruscal-Wallis analysis among ancestral groups. For each ancestral group the synthesis of the TTR expression scores, in terms of median, minimum and maximum, for each tissue involved in TTR amyloidosis is reported. (PDF 124 kb)

Additional file 3: Heatmap of the Dunn’s post-hoc test among the ancestral groups. The colors refer to different significance levels. Detailed information about population definitions is available at http://www.1000genomes.org/about (AFR: Africa, EUR: Europe, EAS: East Asia, SAS: South Asia, AMR: America). (PDF 201 kb)

Additional file 4: Kruscal-Wallis analysis within the European ancestral group. For each population the synthesis of the *TTR* expression scores, in terms of median, minimum and maximum, for each tissue involved in TTR amyloidosis is reported. Detailed information about population definitions is available at http://www.1000genomes.org/about. (PDF 102 kb)

Additional file 5: Kruscal-Wallis analysis within the Eastern Asian ancestral group. For each population the synthesis of the *TTR* expression scores, in terms of median, minimum and maximum, for each tissue involved in TTR amyloidosis is reported. Detailed information about population definitions is available at http://www.1000genomes.org/about. (PDF 101 kb)

Additional file 6: Kruscal-Wallis analysis within the American ancestral group. For each population the synthesis of the *TTR* expression scores, in terms of median, minimum and maximum, for each tissue involved in TTR amyloidosis is reported. Detailed information about population definitions is available at http://www.1000genomes.org/about. (PDF 115 kb)

Additional file 7: Kruscal-Wallis analysis within the African ancestral group. For each population the synthesis of the *TTR* expression scores, in terms of median, minimum and maximum, for each tissue involved in TTR amyloidosis is reported. Detailed information about population definitions is available at http://www.1000genomes.org/about. (PDF 90 kb)

Additional file 8: Kruscal-Wallis analysis within the Southern Asian ancestral group. For each population the synthesis of the *TTR* expression scores, in terms of median, minimum and maximum, for each tissue involved in TTR amyloidosis is reported. Detailed information about population definitions is available at http://www.1000genomes.org/about. (PDF 101 kb)

Additional file 9: List of the selected 132 variants (GRCh37/hg19) with comprehensive information (p-value and effect size) for the 14 investigated tissues. (PDF 138 kb)

Additional file 10: **A**) Clumped datasets for the African ancestry. Colors refer to variants in the LD blocks (r^2^ > 0.5; different colors represent different LD blocks) and X = presence of a variant in a specific dataset after the LD clumping analysis. **B**) Clumped datasets for the European ancestry. Color refer to variants in the LD block (r^2^ > 0.5; different colors represent different LD blocks) and X = presence of a variant in a specific dataset after the LD clumping analysis. **C**) Clumped datasets for the Eastern Asian ancestry. Colors refer to variants in the LD blocks (r^2^ > 0.5; different colors represent different LD blocks) and X = presence of a variant in a specific dataset after the LD clumping analysis **D**) Clumped datasets for the Southern Asian ancestry. Colors refer to variants in the LD blocks (r^2^ > 0.5; different colors represent different LD blocks) and X = presence of a variant in a specific dataset after the LD clumping analysis. **E**) Clumped datasets for the American ancestry. Colors refer to variants in the LD blocks (r^2^ > 0.5; different colors represent different LD blocks) and X = presence of a variant in a specific dataset after the LD clumping analysis. (PDF 88 kb)

## Abbreviations

GTEx: Genotype-Tissue Expression
LD: Linkage Disequilibrium
SNP: Single Nucleotide Polymorphism
TTR: Transthyretin
VCF: Variant Call Format
