# Supplementary material for: Population diversity of the genetically determined TTR expression in human tissues and its implications in TTR amyloidosis
Source: BMC Genomics. 2017 Mar 23;18:254. doi: 10.1186/s12864-017-3646-1 (PMC5364715; doi:10.1186/s12864-017-3646-1)
Supplement: Supplementary file 1 — Inter-ethnic clinical diversity associated with TTR mutations. Each mutation is named in accordance of the missense substitution in the mature protein. For each mutation, rsID and protein change in the protein precursor is also reported. (PDF 137 kb) [file 12864_2017_3646_MOESM1_ESM.pdf]

Additional Data 1: Inter-ethnic clinical diversity associated with TTR mutations. Each mutation is named in accordance of the missense substitution in the mature protein. For each mutation, rsID and protein change in the protein precursor is also reported.

MUTATION (rsID, protein change)	ETHNIC GROUP	CLINCAL DISPLAY	REFERENCES
**Val30Met (rs28933979, p.Val50Met)**	**Europe**	multiform neuropathy, ataxic neuropathy, sensorimotor neuropathy, autonomic neuropathy, nephropathy, oculopathy, asymptomatic, bilateral carpal tunnel, neuropathy, cryptogenic neuropathy, polyneuropathy, gastrointestinal involvement, cardiomyopathy, monoclonal gammopathy, early onset, late onset, urethral injury, incontinence, impotence, proteinuria, diarrhea, constipation, weight loss, peritoneal injury, cranial neuropathy, vitreous opacity, numbness, hypoalgesia and hypoesthesia, muscle injury, autonomic dysfunction, muscle weakness, vomit, cerebral hemorrhage, faint, disautonomy	1-28
	**East Asia**	bilateral carpal tunnel, numbness, dysesthesia, weakness, pulmonary edema, sensorimotor neuropathy, polyneuropathy, dizziness, hepatomegaly, sensorimotor neuropathy, constipation, impotence, diarrhea, orthostatic hypotension, carpal tunnel, cardiomyopathy, oculopathy, dyspnea, nephropathy	29-48
	**Central_South Asia**	numbness, sensorimotor neuropathy, weakness, cardiomyopathy, carpal tunnel, neuropathy, cardiomyopathy	49,50
	**America**	dyspnea, edema, fatigue, paresthesia, cardiomyopathy, carpal tunnel, peripheral neuropathy, polyneuropathy	51-53
**Val30Ala (rs79977247, p.Val50Ala)**	**Europe**	neuropathy, cardiomyopathy	20
	**East Asia**	polyneuropathy, paresthesia, weakness, nausea, vomit, orthostatic hypotension, cardiomyopathy	38,54,55
**Val122Ile (rs76992529, p.Val142Ile)**	**Europe**	polyneuropathy, multiform neuropathy, ataxic neuropathy, motor neuropathy, cardiomyopathy, dyspnea, carpal tunnel	1,56-58,20,28,59

	**Africa**	cardiomyopathy, carpal tunnel, neuropathy, orthostasis, dyspnea, edema, orthopnea, weight loss, late onset, early onset, sleep apnea	60-67
	**America**	cardiomyopathy, bilateral carpal tunnel, peripheral neuropathy, weakness, paresthesia, diarrhea, constipation, impotence, numbness, orthostatic hypotension, incontinence, dyspnea, edema, fatigue, paresthesia	51,68-70
**Ser77Tyr (rs121918071, p.Ser97Tyr)**	**Europe**	polyneuropathy, multiform neuropathy, ataxic neuropathy, motor neuropathy, carpal tunnel, cardiomyopathy	1,28
	**Central_South Asia**	neuropathy, cardiomyopathy	50
**Glu89Lys (p.Glu109Lys)**	**Europe**	cardiomyopathy, oculopathy, vitreous opacity, neuropathy	20,59,71
	**America**	polyneuropathy, cardiomyopathy	72
**Ser50Arg (rs121918076, p.Ser70Arg)**	**Europe**	polyneuropathy, multiform neuropathy, ataxic neuropathy, motor neuropathy, carpal tunnel, cardiomyopathy	1,4,20
	**America**	neuropathy, diarrhea, weight loss, orthostatic hypotension, motor neuropathy, nephropathy, paresthesia, numbness, incontinence, impotence, inappetenza, constipation, weakness, dyspnea	73,74
**Ile107Val (rs121918089, p.Ile127Val)**	**Europe**	polyneuropathy, multiform neuropathy, ataxic neuropathy, motor neuropathy, neuropathy, autonomic dysfunction, muscle weakness	1,22
	**East Asia**	bilater carpal tunnel, weakness, sensorimotor neuropathy, cardiomyopathy, edema, numbness, constipation, muscle atrophy, orthostatic hypotension	75-77
**Phe33Val (p.Phe53Val)**	**Europe**	polyneuropathy, oculopathy, cardiomyopathy, carpal tunnel, vitreous opacity, neuropathy	19,78,20
	**East Asia**	oculopathy, numbness, weight loss, weakness, diarrhea, constipation, nephropathy, muscle atrophy, cardiomyopathy, orthostatic hypotension	79
**Phe33Leu (rs121918068, p.Phe53Leu)**	**Europe**	polyneuropathy, cardiomyopathy, carpal tunnel, diarrhea	10
	**Central_South Asia**	neuropathy	50

**Asp18Gly (rs121918098, p.Asp38Gly)**	**Europe**	memory loss, ataxia, hearing loss, disorientation, vomit, tremors, spastic paresis, hallucinations, urinary retention, constipation, sleep disorders	80
	**East Asia**	impotence, neuropathy, hearing loss, oculopathy, nausea, cardiomyopathy, numbness, leptomeninges involvement	81
**Gly47Glu (p.Gly67Glu)**	**Europe**	cardiomyopathy, autonomic failure, nephropathy, neuropathy	20,59,82,83
	**East Asia**	polyneuropathy, sensorimotor neuropathy, cardiomyopathy, numbness, weakness, constipation, diarrhea, incontinence, impotence	84
**Gly47Ala (rs121918090, Gly67Ala)**	**Europe**	polyneuropathy, multiform neuropathy, ataxic neuropathy, motor neuropathy, cardiomyopathy, axonal neuropathy, carpal tunnel	1,20,85
	**America**	weakness, paresthesia, numbness, weight loss, dyspnea, orthostatic hypotension, diarrhea	74
**Tyr69His (rs121918100, p.Tyr89His)**	**Europe**	oculopathy, neuropathy, paresis, convulsions, vitreous opacity, carpal tunnel	10,86
	**America**	oculopathy, polyneuropathy, leptomeninges involvement, epilepsy, hypertension numbness, nausea, fatigue	87
**Ser23Asn (p.Ser43Asn)**	**Europe**	shortness of breath, chest pain, peripheral edema, cardiomyopathy, neuropathy	20,59,88
	**America**	fatigue, shortness of breath, numbness, cardiomyopathy	89
**Ala25Ser (p.Ala45Ser)**	**Europe**	carpal tunnel, cardiomyopathy, bilateral carpal tunnel, neuropathy	10,86,90
	**America**	polyneuropathy, weakness, fatigue, diarrhea, muscle atrophy, cardiomyopathy	91
**Arg34Gly (p.Arg54Gly)**	**Europe**	carpal tunnel, neuropathy, cardiomyopathy, vitreous opacity	19,92
	**East Asia**	oculopathy	93
**Phe64Ser (rs104894665, p.Phe84Ser)**	**Europe**	carpal tunnel, vitreous opacity, cardiomyopathy	94
	**America**	oculopathy	95

**Thr60Ala (rs121918070, p. Thr80Ala)**	**Europe**	cardiomyopathy, polyneuropathy	96
	**East Asia**	cardiomyopathy, polyneuropathy	97
	**America**	dyspnea, edema, fatigue, paresthesia, cardiomyopathy, peripheral neuropathy	51,52
**Glu54Lys (p.Glu74Lys)**	**Europe**	neuropathy, cardiomyopathy, polyneuropathy, multiform neuropathy, ataxic neuropathy, motor neuropathy	1,20,59
	**East Asia**	diarrhea, constipation, paresthesia, weakness, cardiomyopathy, oculopathy, sensorimotor neuropathy, orthostatic hypotension, polyneuropathy	98
	**Central_South Asia**	diarrhea, numbness, oculopathy, weakness, cardiomyopathy, incontinence, orthostatic hypotension, sensorimotor neuropathy	49
	**America**	paresthesia, oculopathy, diarrhea, orthostatic hypotension, muscle weakness, sensorimotor neuropathy, cardiomyopathy, weight loss	99
**Ala36Pro (rs121918077, p.Ala56Pro)**	**Europe**	vitreous opacity, neuropathy, cardiomyopathy, oculopathy, ataxia, dementia, polyneuropathy	19,20,23
	**East Asia**	oculopathy, paresthesia, weakness, sensorimotor neuropathy, constipation, diarrhea, orthostatic hypotension, cardiomyopathy	100
**Lys35Asn (p.Lys55Asn)**	**Europe**	neuropathy, sensorimotor neuropathy, autonomic dysfunction, muscle weakness	22
	**East Asia**	diarrhea, paresthesia, weakness, orthostatic hypotension, incontinence	101
**Val32Ala (p.Val52Ala)**	**Europe**	neuropathy, sensorimotor neuropathy, autonomic dysfunction, muscle weakness	22
	**Central_South Asia**	polyneuropathy	102
**Leu55Gln (p.Leu75Pro)**	**Europe**	neuropathy, cardiomyopathy, gastrointestinal involvement, diarrhea, constipation, carpal tunnel, glaucoma	86
	**America**	neuropathy, glaucoma, oculopathy	103
**Glu61Lys (rs121918086, p.Glu81Lys)**	**Europe**	polyneuropathy, multiform neuropathy, ataxic neuropathy, motor neuropathy	1

	**East Asia**	dyspnea, numbness, weakness, edema, diarrhea, cardiomyopathy, carpal tunnel, urinary retention, sensorimotor neuropathy, orthostatic hypotension, impotence	104
**Val28Met (p.Val48Met)**	**Europe**	polyneuropathy, multiform neuropathy, ataxic neuropathy, motor neuropathy, impotence, diarrhea, oculopathy, sensorimotor neuropathy, autonomic dysfunction, muscle weakness	1,22,105
**Val30Leu (rs28933979, p.Val50Leu)**	**Europe**	sensorimotor neuropathy, axonal neuropathy, nausea, vomit, diarrhea, cardiomyopathy	86
**Asp38Val (p.Asp58Val)**	**Europe**	cardiomyopathy, sensorimotor neuropathy, disautonomy, gastrointestinal involvement, asymptomatic, bilateral carpal tunnel, dyspnea, pulmonary edema	106
**Asn124Ser (rs144965179, p.Asn144Ser)**	**Europe**	cardiomyopathy	107
**Ser77Phe (p.Ser97Phe)**	**Europe**	polyneuropathy, multiform neuropathy, ataxic neuropathy, motor neuropathy, impotence, muscle injury, autonomic dysfunction	1,22
**His88Arg (p.His108Arg)**	**Europe**	carpal tunnel, cardiomyopathy, nephropathy, constipation, diarrhea, polyneuropathy, axonal neuropathy, dyspnea	10,20,108
**Tyr78Phe (p.Tyr98Phe)**	**Europe**	polyneuropathy, multiform neuropathy, ataxic neuropathy, motor neuropathy, late onset, cardiomyopathy, fatigue, orthostatic hypotension, nephropathy, hepatomegaly, bilateral carpal tunnel, impotence	1,20,109,110
**Glu89Gln (rs121918082, p.Glu109Gln)**	**Europe**	polyneuropathy, multiform neuropathy, ataxic neuropathy, motor neuropathy, disautonomy, hepatomegaly, cardiomyopathy, bilateral carpal tunnel	1,4,20,23
**Arg34Thr (p.Arg54Thr)**	**Europe**	polyneuropathy, disautonomy, neuropathy, cardiomyopathy	4,20
**Thr49Ala (rs121918081, p.Thr69Ala)**	**Europe**	polyneuropathy, multiform neuropathy, ataxic neuropathy, motor neuropathy, disautonomy, neuropathy, cardiomyopathy, vitreous opacity	1,4,19,20

**Thr49Ile (p.Thr69Ile)**	**Europe**	polyneuropathy ,multiform neuropathy, ataxic neuropathy, motor neuropathy, gastrointestinal involvement, cardiomyopathy, weight loss	1,111
**Phe64Leu (rs121918091, p.Phe84Leu)**	**Europe**	polyneuropathy, multiform neuropathy, ataxic neuropathy, motor neuropathy, cryptogenic neuropathy, bilateral carpal tunnel, cardiomyopathy, oculopathy, disautonomy, late onset, asymptomatic, carpal tunnel, orthostatic hypotension, diarrhea, impotence, weight loss, constipation	1,4,14,20,23,59,112
**Phe64Ile (rs121918099, p.Phe84Ile)**	**Europe**	neuropathy, cardiomyopathy	20,59
**Ile107Phe (p.Ile127Phe)**	**Europe**	neuropathy, nephropathy, cardiomyopathy, hepatomegaly	4,20
**Ile68Leu (rs121918085, p.Ile88Leu)**	**Europe**	neuropathy, cardiomyopathy, sensorimotor neuropathy, autonomic neuropathy, polyneuropathy	20,23,28,59,113
**Gly47Arg (p.Gly47Arg)**	**Europe**	polyneuropathy, multiform neuropathy, ataxic neuropathy, motor neuropathy, cardiomyopathy, early onset, impotence, muscle weakness, orthostatic hypotension, diarrhea, muscle atrophy, weight loss	1,20,24
**Val71Ala (rs121918084, p.Val91Ala)**	**Europe**	oculopathy, polyneuropathy, autonomic dysfunction, vitreous opacity, cardiomyopathy, nephropathy, neuropathy, impotence, muscle injury	19,22,114
**Ala39Asp (p.Ala59Asp)**	**Europe**	neuropathy, cardiomyopathy	28
**Gly53Glu (rs121918097, p.Gly73Glu)**	**Europe**	neuropathy, cardiomyopathy, dyspnea	10
**Gly53Ala (p.Gly73Ala)**	**Europe**	neuropathy, cardiomyopathy	20
**His90Asp (p.His110Asp)**	**Europe**	carpal tunnel, numbness, constipation, axonal neuropathy, hepatic involvement	115
**Glu54Val (p.Glu74Val)**	**Europe**	neuropathy	59
**Ala120Ser (p.Ala140Ser)**	**Europe**	neuropathy, cardiomyopathy, nephropathy	14

**Tyr116Ser (rs730881167, p.Tyr116Ser)**	**Europe**	polyneuropathy, multiform neuropathy, ataxic neuropathy, motor neuropathy, neuropathy, bilateral carpal tunnel, autonomic dysfunction, muscle weakness	1,22,116
**Ala91Ser (p.Ala111Ser)**	**Europe**	polyneuropathy, multiform neuropathy, ataxic neuropathy, motor neuropathy, bilateral carpal tunnel, cardiomyopathy	1,116
**Pro24Ser (rs11541790, p.Pro44Ser)**	**Europe**	polyneuropathy, multiform neuropathy, ataxic neuropathy, motor neuropathy	1
**Leu11Met (p.Leu31Met)**	**Europe**	cardiomyopathy	117
**Glu54Gln (p.Glu74Gln)**	**Europe**	polyneuropathy	23
**Gly57Arg (p.Gly77Arg)**	**Europe**	neuropathy, cardiomyopathy, nephropathy, carpal tunnel	20,86,59
**Val32Gly (p.Val52Gly)**	**Europe**	neuropathy, autonomic dysfunction, muscle weakness, polyneuropathy, ataxic neuropathy, motor neuropathy	1,22
**Val14Leu (p.Val34Leu)**	**Europe**	cardiomyopathy	20
**Thr59Lys (rs730881163, p. Thr79Lys)**	**Europe**	neuropathy, cardiomyopathy	20
**Glu92Lys (p.Glu112Lys)**	**Europe**	neuropathy, cardiomyopathy	20
**Cys10Arg (rs121918083, p.Cys30Arg)**	**Europe**	polyneuropathy, multiform neuropathy, ataxic neuropathy, motor neuropathy	1
**Glu42Asp (p.Glu62Asp)**	**Europe**	polyneuropathy, multiform neuropathy, ataxic neuropathy, motor neuropathy	1
**Leu58His (rs121918069, p.Leu78His)**	**Europe**	polyneuropathy, multiform neuropathy, ataxic neuropathy, motor neuropathy	1
**Ile84Asn (p.Ile104Asn)**	**Europe**	polyneuropathy, multiform neuropathy, ataxic neuropathy, motor neuropathy	1
**Val94Ala (p.Val114Ala)**	**Europe**	polyneuropathy, multiform neuropathy, ataxic neuropathy, motor neuropathy	1
**Leu12Pro**	**Africa**	neuropathy, impotence, incontinence, weakness, cardiomyopathy	118

**(rs121918094, p.Leu32Pro)**			
**Asp18Asn (p.Asp38Asn)**	**Africa**	cardiomyopathy, dyspnea, hepatomegaly, edema	119
**Arg104His (rs121918095, p.Arg124His)**	**East Asia**	polyneuropathy	120
**Thr119Met (rs28933981, p.Thr139Met)**	**East Asia**	polyneuropathy	120
**Ala97Ser (rs267607161, p.Ala117Ser)**	**East Asia**	polyneuropathy, numbness, weakness, sensorimotor neuropathy, cardiomyopathy, bilateral carpal tunnel, diarrhea, constipation, pulmonary edema	121,122
**Asp18Glu (rs121918098, p.Asp38Glu)**	**East Asia**	dyspnea, orthostatic hypotension, cardiomyopathy	123
	**America**	cardiomyopathy, bilateral carpal tunnel, weakness, impotence, numbness, edema, oculopathy	124,125
**Asp38Ala (p.Asp58Ala)**	**East Asia**	cardiomyopathy, neuropathy	126
**Gly83Arg (p.Gly103Arg)**	**East Asia**	polyneuropathy, oculopathy, cardiomyopathy	38,127
**Ile73Val (p.Ile93Val)**	**East Asia**	polyneuropathy, gastrointestinal involvement, sensorimotor neuropathy, orthostatic hypotension, cardiomyopathy, incontinence	128
	**Central_South Asia**	polyneuropathy, sensorimotor neuropathy	129
**Lys35Thr (p.Lys55Thr)**	**East Asia**	oculopathy, neuropathy	130
**Leu55Arg (p.Leu75Arg)**	**East Asia**	oculopathy, neuropathy	130
**Ile107Met (p.Ile127Met)**	**East Asia**	polyneuropathy, oculopathy, diarrhea, constipation, sensorimotor neuropathy	131
**Tyr114His (rs121918088, p.Tyr134His)**	**East Asia**	carpal tunnel, bilateral carpal tunnel, polyneuropathy	132,133
**Leu58Arg (p.Leu78Arg)**	**East Asia**	weakness, dysesthesia, sensorimotor neuropathy, impotence, orthostatic hypotension, cardiomyopathy, bilateral carpal tunnel, oculopathy	134,135
**Glu54Gly**	**East Asia**	oculopathy	136

**(p.Glu74Gly)**	**Central_South Asia**	sensorimotor neuropathy, polyneuropathy, nephropathy, dyspnea, cardiomyopathy, oculopathy	137
**Gln92Lys (p.Gln112Lys)**	**East Asia**	cardiomyopathy	138
**Tyr69Ile (p.Tyr89Ile)**	**East Asia**	carpal tunnel, cardiomyopathy	139
**Thr49Ser (p.Tyr69Ser)**	**Central_South Asia**	constipation, bilateral carpal tunnel, cardiomyopathy, orthostatic hypotension, numbness	49
**Ile84Ser (rs121918072, p.Ile104Ser)**	**America**	carpal tunnel, peripheral neuropathy	52
**Ser52Pro (p.Ser72Pro)**	**America**	weakness, paresthesia, numbness, weight loss, dyspnea, orthostatic hypotension, impotence	74
**Thr49Pro (p.Thr69Pro)**	**America**	leptomeninges involvement, polyneuropathy	140
**Val30Gly (rs79977247, p.Val50Gly)**	**America**	oculopathy, shortness of breath, dyspnea, numbness, peripheral neuropathy	141
**Glu61Gly (p.Glu81Gly)**	**America**	cardiomyopathy, fatigue, dyspnea, bilateral carpal tunnel, sleep apnea, edema, sensorial neuropathy	142
**Lys35Thr (p.Lys55Thr)**	**America**	peripheral neuropathy, oculopathy	143
**Trp41Leu (p.Trp61Leu)**	**America**	oculopathy	144

References

1. Adams D, Lozeron P, Theaudin M, et al. Regional difference and similarity of familial amyloidosis with polyneuropathy in France. *Amyloid : the international journal of experimental and clinical investigation : the official journal of the International Society of Amyloidosis.* 2012;19 Suppl 1:61-64.
2. Beirao JM, Moreira LM, Oliveira JC, et al. Aqueous humor erythropoietin levels in open-angle glaucoma patients with and without TTR V30M familial amyloid polyneuropathy. *Molecular vision.* 2014;20:970-976.
3. Buxbaum J, Anan I, Suhr O. Serum transthyretin levels in Swedish TTR V30M carriers. *Amyloid : the international journal of experimental and clinical investigation : the official journal of the International Society of Amyloidosis.* 2010;17(2):83-85.

4. Cappellari M, Cavallaro T, Ferrarini M, et al. Variable presentations of TTR-related familial amyloid polyneuropathy in seventeen patients. *Journal of the peripheral nervous system : JPNS.* 2011;16(2):119-129.
5. Conceicao I. Clinical features of TTR-FAP in Portugal. *Amyloid : the international journal of experimental and clinical investigation : the official journal of the International Society of Amyloidosis.* 2012;19 Suppl 1:71-72.
6. Correia AS, Mendonca M, Caetano A, Medeiros E. A sporadic case of late-onset familial amyloid polyneuropathy with a monoclonal gammopathy. *Neuromuscular disorders : NMD.* 2015;25(8):658-660.
7. Dardiotis E, Koutsou P, Zamba-Papanicolaou E, et al. Complement C1Q polymorphisms modulate onset in familial amyloidotic polyneuropathy TTR Val30Met. *Journal of the neurological sciences.* 2009;284(1-2):158-162.
8. Gomes MJ, Martins Silva A, Salinas Casado J, et al. Is lower urinary tract dysfunction an early marker of Portuguese type familial amyloidotic polyneuropathy in women? Preliminary results. *Archivos espanoles de urologia.* 2014;67(6):557-564.
9. Hellman U, Suhr O. Regional differences and similarities of FAP in Sweden. *Amyloid : the international journal of experimental and clinical investigation : the official journal of the International Society of Amyloidosis.* 2012;19 Suppl 1:53-54.
10. Holmgren G, Hellman U, Lundgren HE, Sandgren O, Suhr OB. Impact of homozygosity for an amyloidogenic transthyretin mutation on phenotype and long term outcome. *Journal of medical genetics.* 2005;42(12):953-956.
11. Iorio A, De Angelis F, Di Girolamo M, et al. Most recent common ancestor of TTR Val30Met mutation in Italian population and its potential role in genotype-phenotype correlation. *Amyloid : the international journal of experimental and clinical investigation : the official journal of the International Society of Amyloidosis.* 2015;22(2):73-78.
12. Lobato L. Portuguese-type amyloidosis (transthyretin amyloidosis, ATTR V30M). *Journal of nephrology.* 2003;16(3):438-442.
13. Lobato L, Beirao I, Silva M, et al. End-stage renal disease and dialysis in hereditary amyloidosis TTR V30M: presentation, survival and prognostic factors. *Amyloid : the international journal of experimental and clinical investigation : the official journal of the International Society of Amyloidosis.* 2004;11(1):27-37.
14. Luigetti M, Conte A, Del Grande A, et al. TTR-related amyloid neuropathy: clinical, electrophysiological and pathological findings in 15 unrelated patients. *Neurological sciences : official journal of the Italian Neurological Society and of the Italian Society of Clinical Neurophysiology.* 2013;34(7):1057-1063.
15. Maia LF, Magalhaes R, Freitas J, et al. CNS involvement in V30M transthyretin amyloidosis: clinical, neuropathological and biochemical findings. *Journal of neurology, neurosurgery, and psychiatry.* 2015;86(2):159-167.
16. Munar-Ques M, Pedrosa JL, Coelho T, et al. Two pairs of proven monozygotic twins discordant for familial amyloid neuropathy (FAP) TTR Met 30. *Journal of medical genetics.* 1999;36(8):629-632.
17. Munar-Ques M, Lopez Dominguez JM, Viader-Farre C, Moreira P, Saraiva MJ. Two Spanish sibs with familial amyloidotic polyneuropathy homozygous for the V30M-TTR gene. *Amyloid : the international journal of experimental and clinical investigation : the official journal of the International Society of Amyloidosis.* 2001;8(2):121-123.

18. Norgren N, Olsson M, Nystrom H, et al. Gene expression profile in hereditary transthyretin amyloidosis: differences in targeted and source organs. *Amyloid : the international journal of experimental and clinical investigation : the official journal of the International Society of Amyloidosis.* 2014;21(2):113-119.
19. Raivio VE, Jonasson J, Myllykangas L, et al. A novel transthyretin Lys70Glu (p.Lys90Glu) mutation presenting with vitreous amyloidosis and carpal tunnel syndrome. *Amyloid : the international journal of experimental and clinical investigation : the official journal of the International Society of Amyloidosis.* 2016;23(1):46-50.
20. Rapezzi C, Quarta CC, Obici L, et al. Disease profile and differential diagnosis of hereditary transthyretin-related amyloidosis with exclusively cardiac phenotype: an Italian perspective. *European heart journal.* 2013;34(7):520-528.
21. Rudolph T, Wilhelm KM, Farbu E. Late-Onset Familial Amyloid Polyneuropathy (FAP) Val30Met Without Family History. *Clinical Medicine & Research.* 2008;6(2):80–82.
22. Said G, Plante-Bordeneuve V. Familial amyloid polyneuropathy: a clinico-pathologic study. *Journal of the neurological sciences.* 2009;284(1-2):149-154.
23. Salvi F, Pastorelli F, Plasmati R, Bartolomei I, Dall'Osso D, Rapezzi C. Genotypic and phenotypic correlation in an Italian population of hereditary amyloidosis TTR-related (HA-TTR): clinical and neurophysiological aids to diagnosis and some reflections on misdiagnosis. *Amyloid : the international journal of experimental and clinical investigation : the official journal of the International Society of Amyloidosis.* 2012;19 Suppl 1:58-60.
24. Salvi F, Pastorelli F, Plasmati R, et al. Brain Microbleeds 12 Years after Orthotopic Liver Transplantation in Val30Met Amyloidosis. *Journal of stroke and cerebrovascular diseases : the official journal of National Stroke Association.* 2015;24(6):e149-151.
25. Seca M, Ferreira N, Coelho T. Vitreous Amyloidosis as the Presenting Symptom of Familial Amyloid Polyneuropathy TTR Val30Met in a Portuguese Patient. *Case reports in ophthalmology.* 2014;5(1):92-97.
26. Soares ML, Coelho T, Sousa A, et al. Haplotypes and DNA sequence variation within and surrounding the transthyretin gene: genotype-phenotype correlations in familial amyloid polyneuropathy (V30M) in Portugal and Sweden. *European journal of human genetics : EJHG.* 2004;12(3):225-237.
27. Soares ML, Coelho T, Sousa A, et al. Susceptibility and modifier genes in Portuguese transthyretin V30M amyloid polyneuropathy: complexity in a single-gene disease. *Human molecular genetics.* 2005;14(4):543-553.
28. Damy T, Costes B, Hagege AA, et al. Prevalence and clinical phenotype of hereditary transthyretin amyloid cardiomyopathy in patients with increased left ventricular wall thickness. *European heart journal.* 2015.
29. Hattori T, Takei Y, Koyama J, Nakazato M, Ikeda S. Clinical and pathological studies of cardiac amyloidosis in transthyretin type familial amyloid polyneuropathy. *Amyloid : the international journal of experimental and clinical investigation : the official journal of the International Society of Amyloidosis.* 2003;10(4):229-239.
30. Ikeda S, Nakazato M, Ando Y, Sobue G. Familial transthyretin-type amyloid polyneuropathy in Japan: clinical and genetic heterogeneity. *Neurology.* 2002;58(7):1001-1007.

31. Kato-Motozaki Y, Ono K, Shima K, et al. Epidemiology of familial amyloid polyneuropathy in Japan: Identification of a novel endemic focus. *Journal of the neurological sciences.* 2008;270(1-2):133-140.
32. Koike H, Sobue G. Late-onset familial amyloid polyneuropathy in Japan. *Amyloid : the international journal of experimental and clinical investigation : the official journal of the International Society of Amyloidosis.* 2012;19 Suppl 1:55-57.
33. Koike H, Misu K, Ikeda S, et al. Type I (transthyretin Met30) familial amyloid polyneuropathy in Japan: early- vs late-onset form. *Archives of neurology.* 2002;59(11):1771-1776.
34. Koike H, Misu K, Sugiura M, et al. Pathology of early- vs late-onset TTR Met30 familial amyloid polyneuropathy. *Neurology.* 2004;63(1):129-138.
35. Koike H, Kawagashira Y, Iijima M, et al. Electrophysiological features of late-onset transthyretin Met30 familial amyloid polyneuropathy unrelated to endemic foci. *Journal of neurology.* 2008;255(10):1526-1533.
36. Koike H, Ando Y, Ueda M, et al. Distinct characteristics of amyloid deposits in early- and late-onset transthyretin Val30Met familial amyloid polyneuropathy. *Journal of the neurological sciences.* 2009;287(1-2):178-184.
37. Koike H, Hashimoto R, Tomita M, et al. Diagnosis of sporadic transthyretin Val30Met familial amyloid polyneuropathy: a practical analysis. *Amyloid : the international journal of experimental and clinical investigation : the official journal of the International Society of Amyloidosis.* 2011;18(2):53-62.
38. Liu T, Zhang B, Jin X, et al. Ophthalmic manifestations in a Chinese family with familial amyloid polyneuropathy due to a TTR Gly83Arg mutation. *Eye.* 2014;28(1):26-33.
39. Miyamura M, Terasaki F, Ishibashi K, et al. Two siblings diagnosed to have transthyretin-related familial amyloid cardiomyopathy around the same time at different hospitals. *Internal medicine.* 2012;51(5):465-469.
40. Sakashita N, Ando Y, Jinnouchi K, et al. Familial amyloidotic polyneuropathy (ATTR Val30Met) with widespread cerebral amyloid angiopathy and lethal cerebral hemorrhage. *Pathology international.* 2001;51(6):476-480.
41. Takahashi R, Ono K, Shibata S, et al. Efficacy of diflunisal on autonomic dysfunction of late-onset familial amyloid polyneuropathy (TTR Val30Met) in a Japanese endemic area. *Journal of the neurological sciences.* 2014;345(1-2):231-235.
42. Takigawa M, Hashimura K, Ishibashi-Ueda H, et al. Annual electrocardiograms consistent with silent progression of cardiac involvement in sporadic familial amyloid polyneuropathy: a case report. *Internal medicine.* 2010;49(2):139-144.
43. Tojo K, Tsuchiya-Suzuki A, Sekijima Y, Morita H, Sumita N, Ikeda S. Upper limb neuropathy such as carpal tunnel syndrome as an initial manifestation of ATTR Val30Met familial amyloid polyneuropathy. *Amyloid : the international journal of experimental and clinical investigation : the official journal of the International Society of Amyloidosis.* 2010;17(1):32-35.
44. Tojo K, Sekijima Y, Machida K, Tsuchiya A, Yazaki M, Ikeda S. Amyloidogenic transthyretin Val30Met homozygote showing unusually early-onset familial amyloid polyneuropathy. *Muscle & nerve.* 2008;37(6):796-803.
45. Ueda M, Ando Y, Haraoka K, et al. Aging and transthyretin-related amyloidosis: pathologic examinations in pulmonary amyloidosis. *Amyloid : the international journal of experimental and clinical investigation : the official journal of the International Society of Amyloidosis.* 2006;13(1):24-30.

46. Araki S, Yi S. Pathology of familial amyloidotic polyneuropathy with TTR met 30 in Kumamoto, Japan. *Neuropathology : official journal of the Japanese Society of Neuropathology.* 2000;20 Suppl:S47-51.
47. Misu K, Hattori N, Ando Y, Ikeda S, Sobue G. Anticipation in early- but not late-onset familial amyloid polyneuropathy (TTR Met 30) in Japan. *Neurology.* 2000:452.
48. Nakamura Y, Yutani C, Nakazato M, Date Y, Baba T, Goto Y. A case of hereditary amyloidosis transthyretin variant Met 30 with amyloid cardiomyopathy, less polyneuropathy, and the presence of giant cells. *Pathology international.* 1999;49(10):898-902.
49. Bekircan-Kurt CE, Gunes N, Yilmaz A, Erdem-Ozdamar S, Tan E. Three Turkish families with different transthyretin mutations. *Neuromuscular disorders : NMD.* 2015;25(9):686-692.
50. Leibou L, Frand J, Sadeh M, et al. Clinical and genetic findings in eight Israeli patients with transthyretin-associated familial amyloid polyneuropathy. *The Israel Medical Association journal : IMAJ.* 2012;14(11):662-665.
51. Arruda-Olson AM, Zeldenrust SR, Dispenzieri A, et al. Genotype, echocardiography, and survival in familial transthyretin amyloidosis. *Amyloid : the international journal of experimental and clinical investigation : the official journal of the International Society of Amyloidosis.* 2013;20(4):263-268.
52. Benson MD, Teague SD, Kovacs R, Feigenbaum H, Jung J, Kincaid JC. Rate of progression of transthyretin amyloidosis. *The American journal of cardiology.* 2011;108(2):285-289.
53. Palacios SA, Bittencourt PL, Cancado EL, et al. Familial amyloidotic polyneuropathy type 1 in Brazil is associated with the transthyretin Val30Met variant. *Amyloid : the international journal of experimental and clinical investigation : the official journal of the International Society of Amyloidosis.* 1999;6(4):289-291.
54. Liu JY, Jiang XM, Zhang M, Guo YJ. Analysis of mitochondrial haplogroups associated with TTR Val30Ala familial amyloidotic polyneuropathy in Chinese patients. *The International journal of neuroscience.* 2012;122(12):716-718.
55. Mak CM, Lam CW, Fan ST, Liu CL, Tam SC. Genetics of familial amyloidotic polyneuropathy in a Hong Kong Chinese kindred. *Acta neurologica Scandinavica.* 2003;107(6):419-422.
56. Ammirati E, Marziliano N, Vittori C, et al. The first Caucasian patient with p.Val122Ile mutated-transthyretin cardiac amyloidosis treated with isolated heart transplantation. *Amyloid : the international journal of experimental and clinical investigation : the official journal of the International Society of Amyloidosis.* 2012;19(2):113-117.
57. Gillmore JD, Booth DR, Pepys MB, Hawkins PN. Hereditary cardiac amyloidosis associated with the transthyretin Ile122 mutation in a white man. *Heart.* 1999;82(3):e2.
58. Hamidi Asl K, Nakamura M, Yamashita T, Benson MD. Cardiac amyloidosis associated with the transthyretin Ile122 mutation in a Caucasian family. *Amyloid : the international journal of experimental and clinical investigation : the official journal of the International Society of Amyloidosis.* 2001;8(4):263-269.
59. Cappelli F, Baldasseroni S, Bergesio F, et al. Echocardiographic and biohumoral characteristics in patients with AL and TTR amyloidosis at diagnosis. *Clinical cardiology.* 2015;38(2):69-75.

60. Connors LH, Prokaeva T, Lim A, et al. Cardiac amyloidosis in African Americans: comparison of clinical and laboratory features of transthyretin V122I amyloidosis and immunoglobulin light chain amyloidosis. *American heart journal.* 2009;158(4):607-614.
61. Hamour IM, Lachmann HJ, Goodman HJ, et al. Heart transplantation for homozygous familial transthyretin (TTR) V122I cardiac amyloidosis. *American journal of transplantation : official journal of the American Society of Transplantation and the American Society of Transplant Surgeons.* 2008;8(5):1056-1059.
62. Jacobson D, Tagoe C, Schwartzbard A, Shah A, Koziol J, Buxbaum J. Relation of clinical, echocardiographic and electrocardiographic features of cardiac amyloidosis to the presence of the transthyretin V122I allele in older African-American men. *The American journal of cardiology.* 2011;108(3):440-444.
63. Jacobson DR, Alexander AA, Tagoe C, Buxbaum JN. Prevalence of the amyloidogenic transthyretin (TTR) V122I allele in 14 333 African-Americans. *Amyloid : the international journal of experimental and clinical investigation : the official journal of the International Society of Amyloidosis.* 2015;22(3):171-174.
64. Molina G, Judge D, Campbell W, Chahal H, Mugmon M. Transthyretin cardiac amyloidosis: an under-diagnosed cause of heart failure. *Journal of Community Hospital Internal Medicine Perspectives.* 2014;4:25500.
65. Patel KS, Hawkins PN. Cardiac amyloidosis: where are we today? *Journal of internal medicine.* 2015;278(2):126-144.
66. Reddi HV, Jenkins S, Theis J, et al. Homozygosity for the V122I mutation in transthyretin is associated with earlier onset of cardiac amyloidosis in the African American population in the seventh decade of life. *The Journal of molecular diagnostics : JMD.* 2014;16(1):68-74.
67. Thenappan T, Fedson S, Rich J, et al. Isolated heart transplantation for familial transthyretin (TTR) V122I cardiac amyloidosis. *Amyloid : the international journal of experimental and clinical investigation : the official journal of the International Society of Amyloidosis.* 2014;21(2):120-123.
68. Carr AS, Pelayo-Negro AL, Jaunmuktane Z, et al. Transthyretin V122I amyloidosis with clinical and histological evidence of amyloid neuropathy and myopathy. *Neuromuscular disorders : NMD.* 2015;25(6):511-515.
69. Quarta CC, Buxbaum JN, Shah AM, et al. The amyloidogenic V122I transthyretin variant in elderly black Americans. *The New England journal of medicine.* 2015;372(1):21-29.
70. Ruberg FL, Maurer MS, Judge DP, et al. Prospective evaluation of the morbidity and mortality of wild-type and V122I mutant transthyretin amyloid cardiomyopathy: the Transthyretin Amyloidosis Cardiac Study (TRACS). *American heart journal.* 2012;164(2):222-228 e221.
71. Sandhu R, Westcott M, Pavesio C, et al. Retinal microangiopathy as an initial manifestation of familial amyloid cardiomyopathy associated with transthyretin e89k mutation. *Retinal cases & brief reports.* 2013;7(3):271-275.
72. Nakamura M, Hamidi Asl K, Benson MD. A novel variant of transthyretin (Glu89Lys) associated with familial amyloidotic polyneuropathy. *Amyloid : the international journal of experimental and clinical investigation : the official journal of the International Society of Amyloidosis.* 2000;7(1):46-50.

73. Gonzalez-Duarte A, Soto KC, Martinez-Banos D, et al. Familial amyloidosis with polyneuropathy associated with TTR Ser50Arg mutation. *Amyloid : the international journal of experimental and clinical investigation : the official journal of the International Society of Amyloidosis.* 2012;19(4):171-176.
74. Gonzalez-Duarte A, Lem-Carrillo M, Cardenas-Soto K. Description of transthyretin S50A, S52P and G47A mutations in familial amyloidosis polyneuropathy. *Amyloid : the international journal of experimental and clinical investigation : the official journal of the International Society of Amyloidosis.* 2013;20(4):221-225.
75. Asahina M, Akaogi Y, Misawa S, et al. Sensorimotor manifestations without autonomic symptoms in two siblings with TTR Val107 familial amyloid polyneuropathy. *Clinical neurology and neurosurgery.* 2011;113(2):139-141.
76. Nanri K, Utsumi H, Yamada M, et al. Transthyretin Val 107 in a Japanese patient with familial amyloid polyneuropathy. *Journal of the neurological sciences.* 2002;198(1-2):93-96.
77. Uotani K, Kawata A, Nagao M, Mizutani T, Hayashi H. Trigger finger as an initial manifestation of familial amyloid polyneuropathy in a patient with Ile107Val TTR. *Internal medicine.* 2007;46(8):501-504.
78. Frigerio R, Fabrizi GM, Ferrarini M, et al. An unusual transthyretin gene missense mutation (TTR Phe33Val) linked to familial amyloidotic polyneuropathy. *Amyloid : the international journal of experimental and clinical investigation : the official journal of the International Society of Amyloidosis.* 2004;11(2):121-124.
79. Kono S, Manabe Y, Tanaka T, et al. A case of familial amyloid polyneuropathy due to Phe33Val TTR with vitreous involvement as the initial manifestation. *Internal medicine.* 2010;49(12):1213-1216.
80. Garzuly F, Vidal R, Wisniewski T, Brittig F, Budka H. Familial meningocerebrovascular amyloidosis, Hungarian type, with mutant transthyretin (TTR Asp18Gly). *Neurology.* 1996;47(6):1562-1567.
81. Jin K, Sato S, Takahashi T, et al. Familial leptomeningeal amyloidosis with a transthyretin variant Asp18Gly representing repeated subarachnoid haemorrhages with superficial siderosis. *Journal of neurology, neurosurgery, and psychiatry.* 2004;75(10):1463-1466.
82. Haagsma EB, Hawkins PN, Benson MD, Lachmann HJ, Bybee A, Hazenberg BP. Familial amyloidotic polyneuropathy with severe renal involvement in association with transthyretin Gly47Glu in Dutch, British and American-Finnish families. *Amyloid : the international journal of experimental and clinical investigation : the official journal of the International Society of Amyloidosis.* 2004;11(1):44-49.
83. Pelo E, Da Prato L, Ciaccheri M, et al. Familial amyloid polyneuropathy with genetic anticipation associated to a gly47glu transthyretin variant in an Italian kindred. *Amyloid : the international journal of experimental and clinical investigation : the official journal of the International Society of Amyloidosis.* 2002;9(1):35-41.
84. Mak CM, Kwong YL, Lam CW, et al. Identification of a novel TTR Gly67Glu mutant and the first case series of familial transthyretin amyloidosis in Hong Kong Chinese. *Amyloid : the international journal of experimental and clinical investigation : the official journal of the International Society of Amyloidosis.* 2007;14(4):293-297.

85. Magy N, Valleix S, Grateau G, et al. Transthyretin mutation (TTRGly47Ala) associated with familial amyloid polyneuropathy in a French family. *Amyloid : the international journal of experimental and clinical investigation : the official journal of the International Society of Amyloidosis.* 2002;9(4):272-275.
86. Suhr OB, Andersen O, Aronsson T, et al. Report of five rare or previously unknown amyloidogenic transthyretin mutations disclosed in Sweden. *Amyloid : the international journal of experimental and clinical investigation : the official journal of the International Society of Amyloidosis.* 2009;16(4):208-214.
87. Schweitzer K, Ehmann D, Garcia R, Alport E. Oculoleptomeningeal amyloidosis in 3 individuals with the transthyretin variant Tyr69His. *Canadian journal of ophthalmology. Journal canadien d'ophtalmologie.* 2009;44(3):317-319.
88. Mueller, II, Gawaz M, Linke RP, et al. Restrictive cardiomyopathy in inherited ATTR amyloidosis (TTR-Ser23Asn) in a patient of German-Italian extraction. *BMJ case reports.* 2010;2010.
89. Castano A, Bokhari S, Brannagan TH, 3rd, Wynn J, Maurer MS. Technetium pyrophosphate myocardial uptake and peripheral neuropathy in a rare variant of familial transthyretin (TTR) amyloidosis (Ser23Asn): a case report and literature review. *Amyloid : the international journal of experimental and clinical investigation : the official journal of the International Society of Amyloidosis.* 2012;19(1):41-46.
90. Janunger T, Anan I, Holmgren G, et al. Heart failure caused by a novel amyloidogenic mutation of the transthyretin gene: ATTR Ala45Ser. *Amyloid : the international journal of experimental and clinical investigation : the official journal of the International Society of Amyloidosis.* 2000;7(2):137-140.
91. Yazaki M, Yamashita T, Kincaid JC, et al. Rapidly progressive amyloid polyneuropathy associated with a novel variant transthyretin serine 25. *Muscle & nerve.* 2002;25(2):244-250.
92. Levy J, Hawkins PN, Rowczenio D, Godfrey T, Stawell R, Zamir E. Familial amyloid polyneuropathy associated with the novel transthyretin variant Arg34Gly. *Amyloid : the international journal of experimental and clinical investigation : the official journal of the International Society of Amyloidosis.* 2012;19(4):201-203.
93. Shi Y, Li J, Hu J, et al. A new Arg54Gly transthyretin gene mutation associated with vitreous amyloidosis in Chinese. *Eye science.* 2011;26(4):230-238.
94. Zolyomi Z, Benson MD, Halasz K, Uemichi T, Fekete G. Transthyretin mutation (serine 84) associated with familial amyloid polyneuropathy in a Hungarian family. *Amyloid : the international journal of experimental and clinical investigation : the official journal of the International Society of Amyloidosis.* 1998;5(1):30-34.
95. Naderi AS, Farsian FN, Igarashi P. Gastrointestinal amyloidosis associated with transthyretin Phe64Ser mutation. *The American journal of the medical sciences.* 2007;334(3):219-221.
96. Sattianayagam PT, Hahn AF, Whelan CJ, et al. Cardiac phenotype and clinical outcome of familial amyloid polyneuropathy associated with transthyretin alanine 60 variant. *European heart journal.* 2012;33(9):1120-1127.

97. Kotani N, Hattori T, Yamagata S, et al. Transthyretin Thr60Ala Appalachian-type mutation in a Japanese family with familial amyloidotic polyneuropathy. *Amyloid : the international journal of experimental and clinical investigation : the official journal of the International Society of Amyloidosis.* 2002;9(1):31-34.
98. Nagasaka T, Togashi S, Watanabe H, et al. Clinical and histopathological features of progressive-type familial amyloidotic polyneuropathy with TTR Lys54. *Journal of the neurological sciences.* 2009;276(1-2):88-94.
99. Busse A, Sanchez MA, Monterroso V, Alvarado MV, Leon P. A severe form of amyloidotic polyneuropathy in a Costa Rican family with a rare transthyretin mutation (Glu54Lys). *American journal of medical genetics. Part A.* 2004;128A(2):190-194.
100. Zou X, Dong F, Zhang S, Tian R, Sui R. Transthyretin Ala36Pro mutation in a Chinese pedigree of familial transthyretin amyloidosis with elevated vitreous and serum vascular endothelial growth factor. *Experimental eye research.* 2013;110:44-49.
101. Ryu JK, Baik HW, Bae JS, et al. Familial amyloid polyneuropathy in Korea: the first case report with a proven ATTR Lys35Asn gene. *Amyloid : the international journal of experimental and clinical investigation : the official journal of the International Society of Amyloidosis.* 2005;12(1):62-64.
102. Kaplan B, Shinar Y, Avisar C, Livneh A. Transthyretin amyloidosis in a patient of Iranian-Jewish extraction: a second Israeli-Jewish case. *Clinical chemistry and laboratory medicine.* 2007;45(5):625-628.
103. Yazaki M, Varga J, Dyck PJ, Benson MD. A new transthyretin variant Leu55Gln in a patient with systemic amyloidosis. *Amyloid : the international journal of experimental and clinical investigation : the official journal of the International Society of Amyloidosis.* 2002;9(4):268-271.
104. Noto Y, Tokuda T, Shiga K, et al. Cardiomyopathy in a Japanese family with the Glu61Lys transthyretin variant: a new phenotype. *Amyloid : the international journal of experimental and clinical investigation : the official journal of the International Society of Amyloidosis.* 2009;16(2):99-102.
105. de Carvalho M, Moreira P, Evangelista T, et al. New transthyretin mutation V28M in a Portuguese kindred with amyloid polyneuropathy. *Muscle & nerve.* 2000;23(7):1016-1021.
106. Augustin S, Llige D, Andreu A, Gonzalez A, Genesca J. Familial amyloidosis in a large Spanish kindred resulting from a D38V mutation in the transthyretin gene. *European journal of clinical investigation.* 2007;37(8):673-678.
107. Bergstrom J, Patrosso MC, Colussi G, et al. A novel type of familial transthyretin amyloidosis, ATTR Asn124Ser, with co-localization of kappa light chains. *Amyloid : the international journal of experimental and clinical investigation : the official journal of the International Society of Amyloidosis.* 2007;14(2):141-145.
108. Hellman U, Lundgren HE, Westermark P, et al. A genealogical and clinical study of the phenotypical variation within the Swedish transthyretin His88Arg (p. His108Arg) amyloidosis family. *European journal of medical genetics.* 2015;58(4):211-215.
109. Magy N, Liepnieks JJ, Gil H, et al. A transthyretin mutation (Tyr78Phe) associated with peripheral neuropathy, carpal tunnel syndrome and skin amyloidosis. *Amyloid : the international journal of experimental and clinical investigation : the official journal of the International Society of Amyloidosis.* 2003;10(1):29-33.

110. Riboldi G, Del Bo R, Ranieri M, et al. Tyr78Phe Transthyretin Mutation with Predominant Motor Neuropathy as the Initial Presentation. *Case reports in neurology.* 2011;3(1):62-68.
111. Saraiva MJ, Munar-Ques M, Modrego P, Moreira P, Viader-Farre C. First Spanish family with familial amyloidotic polyneuropathy associated to TTR Thr49Ile mutation. *Amyloid : the international journal of experimental and clinical investigation : the official journal of the International Society of Amyloidosis.* 2003;10(1):34-35.
112. Russo M, Mazzeo A, Stancanelli C, et al. Transthyretin-related familial amyloidotic polyneuropathy: description of a cohort of patients with Leu64 mutation and late onset. *Journal of the peripheral nervous system : JPNS.* 2012;17(4):385-390.
113. Salvi F, Scaglione C, Michelucci R, et al. Atypical familial motor neuropathy in patients with mutant TTR Ile68Leu. *Amyloid : the international journal of experimental and clinical investigation : the official journal of the International Society of Amyloidosis.* 2003;10(3):185-189.
114. Haagsma EB, Scheffer H, Altland K, De Jager AE, Hazenberg BP. Transthyretin Val71Ala mutation in a Dutch family with familial amyloidotic polyneuropathy. *Amyloid : the international journal of experimental and clinical investigation : the official journal of the International Society of Amyloidosis.* 2000;7(3):218-221.
115. Jimenez-Zepeda VH, Bahlis NJ, Gilbertson J, et al. A novel transthyretin variant p.H110D (H90D) as a cause of familial amyloid polyneuropathy in a large Irish kindred. *Amyloid : the international journal of experimental and clinical investigation : the official journal of the International Society of Amyloidosis.* 2015;22(1):26-30.
116. Misrahi AM, Plante V, Lalu T, et al. New transthyretin variants SER 91 and SER 116 associated with familial amyloidotic polyneuropathy. Mutations in brief no. 151. Online. *Human mutation.* 1998;12(1):71.
117. Nelson LM, Penninga L, Villadsen GE, et al. Outcome in patients treated with isolated liver transplantation for familial transthyretin amyloidosis to prevent cardiomyopathy. *Clinical transplantation.* 2015;29(12):1098-1104.
118. McColgan P, Viegas S, Gandhi S, et al. Oculoleptomeningeal Amyloidosis associated with transthyretin Leu12Pro in an African patient. *Journal of neurology.* 2015;262(1):228-234.
119. Quarta CC, Falk RH. A transthyretin variant, Asp18Asn, associated with amyloid cardiomyopathy: a new African-American variant? *Amyloid : the international journal of experimental and clinical investigation : the official journal of the International Society of Amyloidosis.* 2012;19(4):204-207.
120. Almeida MR, Alves IL, Terazaki H, Ando Y, Saraiva MJ. Comparative studies of two transthyretin variants with protective effects on familial amyloidotic polyneuropathy: TTR R104H and TTR T119M. *Biochemical and biophysical research communications.* 2000;270(3):1024-1028.
121. Hsieh ST. Amyloid neuropathy with transthyretin mutations: overview and unique Ala97Ser in Taiwan. *Acta neurologica Taiwanica.* 2011;20(2):155-160.
122. Yang NC, Lee MJ, Chao CC, et al. Clinical presentations and skin denervation in amyloid neuropathy due to transthyretin Ala97Ser. *Neurology.* 2010;75(6):532-538.

123. Imamura T, Nakazato M, Date Y, et al. Cardiac amyloidosis associated with a novel transthyretin aspartic acid-18 glutamic acid de novo mutation. *Circulation journal : official journal of the Japanese Circulation Society.* 2003;67(11):965-968.
124. Connors LH, Yamashita T, Yazaki M, Skinner M, Benson MD. A rare transthyretin mutation (Asp18Glu) associated with cardiomyopathy. *Amyloid : the international journal of experimental and clinical investigation : the official journal of the International Society of Amyloidosis.* 2004;11(1):61-66.
125. Solano JM, Pulido JS, Salomao DR. A rare transthyretin mutation (Asp18Glu) associated with vitreous amyloid. *Ophthalmic genetics.* 2007;28(2):73-75.
126. Jang MA, Lee GY, Kim K, et al. Asp58Ala is the predominant mutation of the TTR gene in Korean patients with hereditary transthyretin-related amyloidosis. *Annals of human genetics.* 2015;79(2):99-107.
127. Zhang AM, Wang H, Sun P, Hu QX, He Y, Yao YG. Mutation p.G83R in the transthyretin gene is associated with hereditary vitreous amyloidosis in Han Chinese families. *Molecular vision.* 2013;19:1631-1638.
128. Liao MF, Chang HS. A novel variant mutation of transthyretin Ile73Val-related amyloidotic polyneuropathy in Taiwanese. *Acta neurologica Taiwanica.* 2013;22(2):87-92.
129. Booth DR, Gillmore JD, Persey MR, et al. Transthyretin Ile73Val is associated with familial amyloidotic polyneuropathy in a Bangladeshi family. Mutations in brief no. 158. Online. *Human mutation.* 1998;12(2):135.
130. Long D, Zeng J, Wu LQ, Tang LS, Wang HL, Wang H. Vitreous amyloidosis in two large mainland Chinese kindreds resulting from transthyretin variant Lys35Thr and Leu55Arg. *Ophthalmic genetics.* 2012;33(1):28-33.
131. Lv W, Chen J, Chen W, Hou P, Pang CP, Chen H. Multimodal retinal imaging in a Chinese kindred with familial amyloid polyneuropathy secondary to transthyretin Ile107Met mutation. *Eye.* 2014;28(4):452-458.
132. Mochizuki H, Kamakura K, Masaki T, et al. Nodular cutaneous amyloidosis and carpal tunnel syndrome due to the amyloidogenic transthyretin His 114 variant. *Amyloid : the international journal of experimental and clinical investigation : the official journal of the International Society of Amyloidosis.* 2001;8(2):105-110.
133. Sekijima Y, Tojo K, Morita H, Koyama J, Ikeda S. Safety and efficacy of long-term diflunisal administration in hereditary transthyretin (ATTR) amyloidosis. *Amyloid : the international journal of experimental and clinical investigation : the official journal of the International Society of Amyloidosis.* 2015;22(2):79-83.
134. Motozaki Y, Sugiyama Y, Ishida C, Komai K, Matsubara S, Yamada M. Phenotypic heterogeneity in a family with FAP due to a TTR Leu58Arg mutation: a clinicopathologic study. *Journal of the neurological sciences.* 2007;260(1-2):236-239.
135. Togashi S, Watanabe H, Nagasaka T, et al. An aggressive familial amyloidotic polyneuropathy caused by a new variant transthyretin Lys 54. *Neurology.* 1999;53(3):637-639.
136. O'Hearn TM, Fawzi A, He S, Rao NA, Lim JI. Early onset vitreous amyloidosis in familial amyloidotic polyneuropathy with a transthyretin Glu54Gly mutation is associated with elevated vitreous VEGF. *The British journal of ophthalmology.* 2007;91(12):1607-1609.

137. Schanzer A, Kimmich C, Rocken C, et al. A woman with a rare p.Glu74Gly transthyretin mutation presenting exclusively with a rapidly progressive neuropathy: a case report. *Journal of medical case reports.* 2014;8:403.
138. Saito F, Nakazato M, Akiyama H, et al. A case of late onset cardiac amyloidosis with a new transthyretin variant (lysine 92). *Human pathology.* 2001;32(2):237-239.
139. Takei Y, Hattori T, Yazaki M, et al. Transthyretin Tyr69-to-Ile mutation (double-nucleotide substitution in codon 69) in a Japanese familial amyloidosis patient with cardiomyopathy and carpal tunnel syndrome. *Amyloid : the international journal of experimental and clinical investigation : the official journal of the International Society of Amyloidosis.* 2003;10(1):25-28.
140. Nakagawa K, Sheikh SI, Snuderl M, Frosch MP, Greenberg SM. A new Thr49Pro transthyretin gene mutation associated with leptomeningeal amyloidosis. *Journal of the neurological sciences.* 2008;272(1-2):186-190.
141. Roe RH, Fisher Y, Eagle RC, Jr., Fine HF, Cunningham ET, Jr. Oculoleptomeningeal amyloidosis in a patient with a TTR Val30Gly mutation in the transthyretin gene. *Ophthalmology.* 2007;114(11):e33-37.
142. Rosenzweig M, Skinner M, Prokaeva T, et al. A new transthyretin variant (Glu61Gly) associated with cardiomyopathy. *Amyloid : the international journal of experimental and clinical investigation : the official journal of the International Society of Amyloidosis.* 2007;14(1):65-71.
143. Ruzhansky K, Scoon J, Weimer LH, Maurer MS, Berk JL, Brannagan TH, 3rd. Discordant phenotype in monozygotic female twins with Lys35Thr TTR familial amyloidotic polyneuropathy. *Journal of clinical neuromuscular disease.* 2014;16(1):1-6.
144. Yazaki M, Connors LH, Eagle RC, Jr., Leff SR, Skinner M, Benson MD. Transthyretin amyloidosis associated with a novel variant (Trp41Leu) presenting with vitreous opacities. *Amyloid : the international journal of experimental and clinical investigation : the official journal of the International Society of Amyloidosis.* 2002;9(4):263-267.
