# Supplementary material for: Population diversity of the genetically determined TTR expression in human tissues and its implications in TTR amyloidosis
Source: BMC Genomics. 2017 Mar 23;18:254. doi: 10.1186/s12864-017-3646-1 (PMC5364715; doi:10.1186/s12864-017-3646-1)
Supplement: Supplementary file 2 — Kruscal-Wallis analysis among ancestral groups. For each ancestral group the synthesis of the TTR expression scores, in terms of median, minimum and maximum, for each tissue involved in TTR amyloidosis is reported. (PDF 124 kb) [file 12864_2017_3646_MOESM2_ESM.pdf]

Additional Data 2: Kruscal-Wallis analysis among ancestral groups. For each ancestral group the synthesis of the *TTR* expression scores, in terms of median, minimum and maximum, for each tissue involved in TTR amyloidosis is reported.

	AFRICA			EUROPE			EAST_ASIA			SOUTH_ASIA			AMERICA			
	min	*median*	*max*	*min*	*median*	*max*	*min*	*median*	*max*	*min*	*median*	*max*	*min*	*median*	*max*	**Kruskal-Wallis p-value**
**Colon-Transverse**	-0.22	-0.11	0.30	-0.28	-0.11	0.15	-0.22	-0.06	0.00	-0.22	-0.06	0.04	-0.22	-0.06	0.15	5.448E-11*
**Colon-Sigmoid**	0.00	0.00	0.52	0.00	0.00	0.52	0.00	0.00	0.52	0.00	0.00	0.52	0.00	0.00	0.52	1.8339E-17*
**Esophagus-Muscularis**	0.00	0.00	0.23	-0.32	0.00	0.23	0.00	0.00	0.00	0.00	0.00	0.46	-0.32	0.00	0.23	0.4912
**Esophagus-Mucosa**	0.00	0.10	0.29	0.00	0.00	0.38	0.00	0.10	0.38	0.00	0.00	0.38	0.00	0.00	0.38	1.3638E-58*
**Heart - Atrial Appendage**	-0.60	0.00	0.88	0.00	0.00	0.05	-0.45	0.00	0.02	-0.60	0.00	0.05	-0.60	0.00	0.88	4.5521E-28*
**Heart - Left Ventricle**	-0.86	0.00	0.95	0.00	0.00	0.10	-0.43	0.00	0.06	-0.68	0.00	0.13	-0.60	0.00	0.86	6.5427E-35*
**Liver**	-0.83	0.00	0.11	-0.09	0.00	0.23	-0.58	0.00	0.00	-0.83	0.00	0.00	-0.83	0.00	0.11	7.0173E-66*
**Muscle - Skeletal**	-0.09	-0.09	0.32	-0.09	-0.04	0.17	-0.09	-0.04	0.32	-0.09	0.00	0.33	-0.09	-0.04	0.32	5.0174E-105*
**Nerve-Tibial**	-0.16	-0.08	0.00	-0.16	-0.08	0.32	-0.16	-0.08	0.00	-0.16	-0.08	0.32	-0.16	-0.08	0.00	2.8912E-09*
**Stomach**	-0.31	0.00	0.00	-0.24	0.00	0.00	-0.08	0.00	0.00	-0.16	0.00	0.00	-0.31	0.00	0.00	1.4815E-100*
**Small Intestine - Terminal Ileum**	-0.42	-0.21	0.31	-0.21	0.00	0.63	0.00	0.00	0.31	-0.21	0.00	0.63	-0.42	0.00	0.31	2.0985E-203*
**Adipose - Subcutaneous**	-0.94	0.00	0.00	-0.50	0.00	0.00	-0.94	0.00	0.00	-0.50	0.00	0.00	-0.50	0.00	0.00	6.2184E-11*
**Cells - Transformed fibroblasts**	-0.16	0.00	0.20	-0.10	0.00	0.29	-0.20	0.00	0.20	-0.10	0.00	0.20	-0.07	0.04	0.24	1.8783E-25*
**Skin - Sun Exposed (Lower leg)**	-0.09	0.00	0.57	-0.09	0.00	0.21	-0.07	0.00	0.21	-0.07	0.00	0.10	-0.07	0.00	0.51	1.0020E-86*
**N samples**	661			503			504			489			347			

** p < 0.0035, according to Bonferroni adjustment*
