# Supplementary material for: Population diversity of the genetically determined TTR expression in human tissues and its implications in TTR amyloidosis
Source: BMC Genomics. 2017 Mar 23;18:254. doi: 10.1186/s12864-017-3646-1 (PMC5364715; doi:10.1186/s12864-017-3646-1)
Supplement: Supplementary file 3 — Heatmap of the Dunn’s post-hoc test among the ancestral groups. The colors refer to different significance levels. Detailed information about population definitions is available at http://www.1000genomes.org/about (AFR: Africa, EUR: Europe, EAS: East Asia, SAS: South Asia, AMR: America). (PDF 201 kb) [file 12864_2017_3646_MOESM3_ESM.pdf]

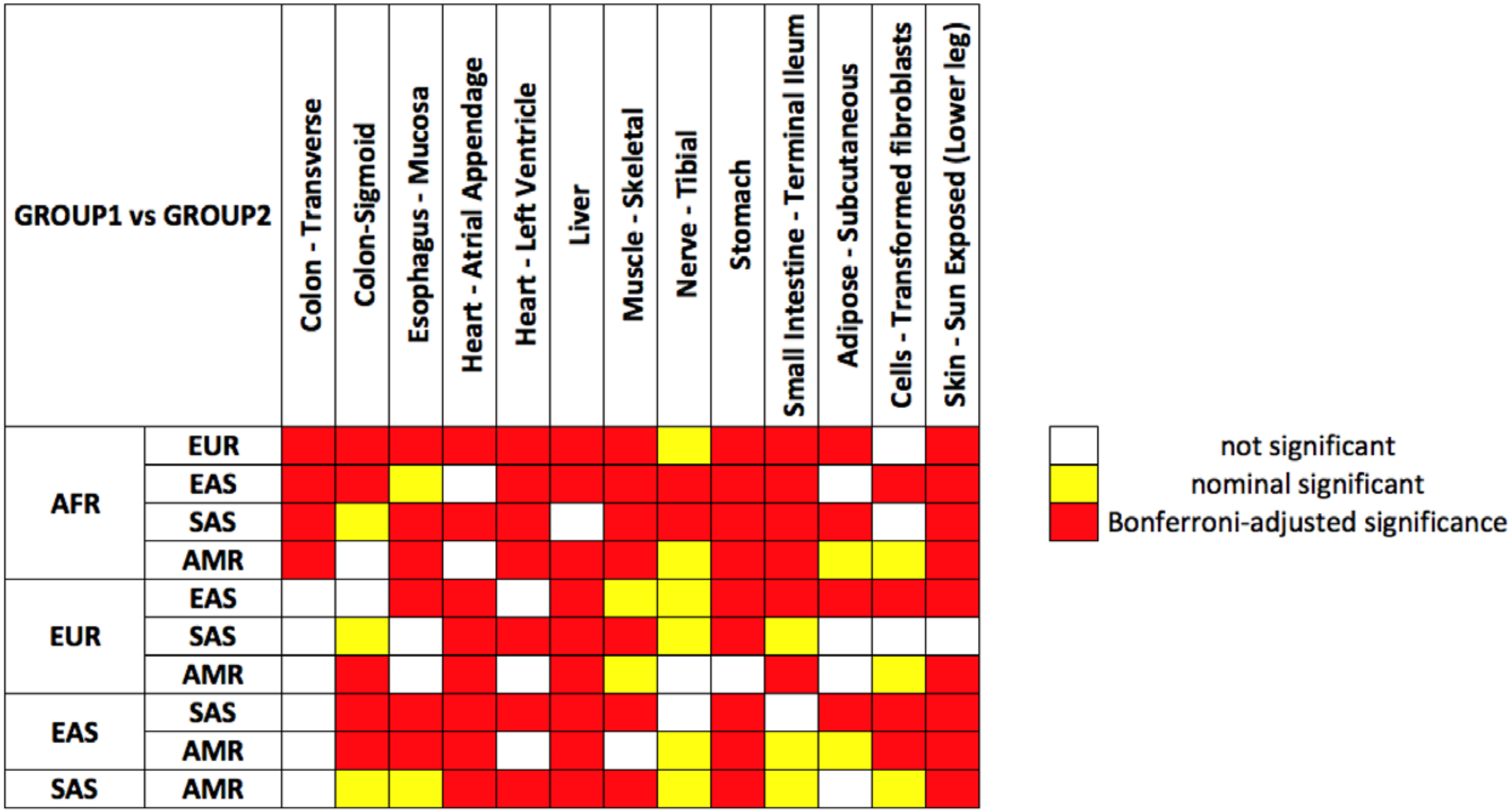

Additional Data 3: Heatmap of the Dunn's post-hoc test among the ancestral groups. The colors refer to different significance levels. Detailed information about population definitions is available at *http://www.1000genomes.org/about.* (AFR: Africa, EUR: Europe, EAS: East Asia, SAS: South Asia, AMR: America).
