# Supplementary material for: Population diversity of the genetically determined TTR expression in human tissues and its implications in TTR amyloidosis
Source: BMC Genomics. 2017 Mar 23;18:254. doi: 10.1186/s12864-017-3646-1 (PMC5364715; doi:10.1186/s12864-017-3646-1)
Supplement: Supplementary file 4 — Kruscal-Wallis analysis within the European ancestral group. For each population the synthesis of the TTR expression scores, in terms of median, minimum and maximum, for each tissue involved in TTR amyloidosis is reported. Detailed information about population definitions is available at http://www.1000genomes.org/about. (PDF 102 kb) [file 12864_2017_3646_MOESM4_ESM.pdf]

Additional Data 4: Kruscal-Wallis analysis within the European ancestral group. For each population the synthesis of the *TTR* expression scores, in terms of median, minimum and maximum, for each tissue involved in TTR amyloidosis is reported. Detailed information about population definitions is available at *http://www.1000genomes.org/about.*

TISSUE	CEU			FIN			GBR			IBS			TSI			Kruskal-Wallis p-value
	min	*median*	*max*	*min*	*median*	*max*	*min*	*median*	*max*	*min*	*median*	*max*	*min*	*median*	*max*	
**Colon-Transverse**	-0.14	0.00	0.13	-0.05	0.00	0.07	-0.16	-0.02	0.13	-0.05	-0.02	0.07	-0.16	-0.02	0.56	0.0024*
**Colon-Sigmoid**	0.00	0.00	0.52	0.00	0.00	0.26	0.00	0.00	0.52	0.00	0.00	0.52	0.00	0.00	0.52	0.2923
**Esophagus-Muscularis**	-0.34	0.00	0.15	0.00	0.00	0.15	-0.34	0.00	0.15	-0.34	0.00	0.15	-0.34	0.00	0.00	0.5622
**Esophagus-Mucosa**	0.00	0.00	0.38	0.00	0.00	0.38	0.00	0.00	0.29	0.00	0.00	0.38	0.00	0.00	0.38	0.3718
**Heart - Atrial Appendage**	0.00	0.00	0.02	0.00	0.00	0.02	0.00	0.00	0.04	0.00	0.00	0.04	-0.04	0.00	0.04	0.0055
**Heart - Left Ventricle**	0.00	0.00	0.07	0.00	0.00	0.07	0.00	0.00	0.07	0.00	0.00	0.07	-0.06	0.00	0.07	0.5261
**Liver**	0.00	0.00	0.11	0.00	0.00	0.11	0.00	0.00	0.23	-0.09	0.00	0.11	-0.09	0.00	0.11	0.2178
**Muscle - Skeletal**	-0.13	-0.07	0.00	-0.13	-0.07	0.00	-0.13	-0.07	0.00	-0.13	-0.07	0.00	-0.13	-0.07	0.00	6.7949E-06*
**Nerve-Tibial**	-0.16	-0.08	0.32	-0.16	-0.08	0.32	-0.16	-0.08	0.24	-0.16	-0.08	0.32	-0.16	-0.08	0.00	0.0359
**Stomach**	-0.08	0.00	0.00	-0.08	0.00	0.00	-0.16	0.00	0.00	-0.24	0.00	0.00	-0.16	0.00	0.00	0.0039
**Small Intestine - Terminal Ileum**	0.00	0.00	0.63	0.00	0.00	0.63	0.00	0.00	0.63	-0.21	0.00	0.63	0.00	0.00	0.31	0.9489
**Adipose - Subcutaneous**	0.00	0.00	0.00	0.00	0.00	0.00	-0.47	0.00	0.00	-0.50	0.00	0.00	-0.50	0.00	0.00	0.7008
**Cells - Transformed fibroblasts**	-0.07	0.00	0.20	-0.07	0.00	0.14	-0.10	0.00	0.29	-0.07	0.00	0.24	-0.07	0.00	0.20	0.0251
**Skin - Sun Exposed (Lower leg)**	-0.08	0.00	0.28	-0.06	0.00	0.14	-0.08	0.00	0.28	-0.06	0.00	0.22	-0.06	0.00	0.28	0.4988
**N samples**	99			99			91			107			107			

** $p < 0.0035$, according to Bonferroni adjustment*
