# Supplementary material for: Population diversity of the genetically determined TTR expression in human tissues and its implications in TTR amyloidosis
Source: BMC Genomics. 2017 Mar 23;18:254. doi: 10.1186/s12864-017-3646-1 (PMC5364715; doi:10.1186/s12864-017-3646-1)
Supplement: Supplementary file 9 — List of the selected 132 variants (GRCh37/hg19) with comprehensive information (p-value and effect size) for the 14 investigated tissues. (PDF 138 kb) [file 12864_2017_3646_MOESM9_ESM.pdf]

**Additional Data 10:** List of the selected 132 variants (GRCh37/hg19) with comprehensive information (p-value and effect size) for the 14 investigated tissues.

| RSID | Location | | Colon - Transverse | Esophagus - Muscularis | Heart - Atrial Appendage | Heart - Left Ventricle | Liver | Muscle - Skeletal | Nerve - Tibial | Stomach | Adipose - Subcutaneous | Cells - Transformed fibroblasts | Skin - Sun Exposed (Lower leg) | Colon - Sigmoid | Esophagus - Mucosa | Small Intestine - Terminal Ileum |
|---|---|---|---|---|---|---|---|---|---|---|---|---|---|---|---|---|
| rs8090089 | upstream | *P-Value* | 0.084 | 0.14 | 0.76 | 0.14 | 0.91 | 0.31 | 0.84 | 0.64 | 0.44 | 0.21 | 0.5 | 0.45 | 0.32 | 0.97 |
| | | *Effect Size* | 0.8 | 0.35 | 0.11 | -0.79 | -0.027 | -0.31 | -0.062 | -0.12 | -0.22 | -0.35 | -0.21 | 0.47 | 0.52 | 0.022 |
| rs4799579 | upstream | *P-Value* | 0.082 | 0.57 | 0.14 | 0.34 | 0.92 | 0.24 | 0.21 | 0.87 | 0.42 | 0.91 | 0.32 | 0.97 | 0.82 | 0.82 |
| | | *Effect Size* | -0.16 | -0.043 | 0.15 | 0.11 | 0.0085 | -0.089 | 0.13 | -0.0088 | 0.065 | 0.009 | -0.077 | 0.0044 | 0.022 | 0.04 |
| rs17660933 | upstream | *P-Value* | 0.16 | 0.95 | 0.12 | 0.54 | 0.95 | 0.18 | 0.2 | 0.78 | 0.56 | 0.8 | 0.23 | 0.88 | 0.7 | 0.81 |
| | | *Effect Size* | -0.13 | -0.0043 | 0.15 | 0.066 | 0.005 | -0.1 | 0.13 | -0.015 | 0.045 | -0.02 | -0.091 | 0.015 | 0.038 | 0.041 |
| rs11081702 | upstream | *P-Value* | 0.16 | 0.96 | 0.11 | 0.54 | 0.94 | 0.18 | 0.21 | 0.78 | 0.56 | 0.8 | 0.23 | 0.89 | 0.7 | 0.83 |
| | | *Effect Size* | -0.13 | -0.004 | 0.15 | 0.066 | 0.0054 | -0.1 | 0.13 | -0.015 | 0.045 | -0.02 | -0.092 | 0.014 | 0.037 | 0.038 |
| rs1667238 | upstream | *P-Value* | 0.25 | 0.44 | 0.11 | 0.68 | 0.14 | 0.14 | 0.42 | 0.77 | 0.37 | 0.82 | 0.53 | 0.84 | 0.49 | 0.44 |
| | | *Effect Size* | 0.39 | -0.19 | -0.53 | -0.43 | -0.61 | 0.45 | 0.25 | 0.099 | 0.28 | 0.055 | -0.16 | -0.17 | -0.23 | 0.79 |
| rs2034404 | upstream | *P-Value* | 0.08 | 0.6 | 0.13 | 0.35 | 0.92 | 0.27 | 0.17 | 0.86 | 0.42 | 0.92 | 0.29 | 0.99 | 0.85 | 0.82 |
| | | *Effect Size* | -0.16 | -0.04 | 0.15 | 0.1 | 0.0082 | -0.084 | 0.14 | -0.0099 | 0.064 | 0.0081 | -0.081 | 0.0011 | 0.019 | 0.04 |
| rs1667239 | upstream | *P-Value* | 0.11 | 0.67 | 0.26 | 0.57 | 0.94 | 0.19 | 0.39 | 0.54 | 0.73 | 0.57 | 0.27 | 0.92 | 0.62 | 0.97 |
| | | *Effect Size* | -0.14 | -0.032 | 0.11 | 0.061 | 0.0058 | -0.099 | 0.084 | -0.032 | 0.027 | -0.043 | -0.083 | -0.01 | 0.047 | -0.0065 |
| rs1375445 | upstream | *P-Value* | 0.099 | 0.67 | 0.29 | 0.63 | 0.94 | 0.18 | 0.41 | 0.53 | 0.74 | 0.57 | 0.27 | 0.94 | 0.62 | 1 |
| | | *Effect Size* | -0.14 | -0.032 | 0.1 | 0.051 | 0.0057 | -0.1 | 0.08 | -0.033 | 0.026 | -0.043 | -0.083 | -0.0077 | 0.047 | -0.00081 |
| rs1791191 | upstream | *P-Value* | 0.88 | 0.1 | 0.064 | 0.068 | 0.088 | 0.87 | 0.75 | 0.82 | 0.34 | 0.51 | 0.8 | 0.89 | 0.49 | 0.52 |
| | | *Effect Size* | -0.068 | -0.46 | -0.6 | -1.2 | -0.6 | 0.052 | 0.11 | 0.059 | 0.32 | -0.18 | -0.069 | 0.09 | -0.27 | 0.38 |
| rs1375446 | upstream | *P-Value* | 0.19 | 0.59 | 0.27 | 0.54 | 0.67 | 0.23 | 0.37 | 0.45 | 0.78 | 0.45 | 0.52 | 0.7 | 0.41 | 0.8 |
| | | *Effect Size* | -0.12 | -0.041 | 0.11 | 0.065 | 0.031 | -0.093 | 0.09 | -0.041 | 0.022 | -0.059 | -0.048 | 0.039 | 0.077 | 0.04 |

| | | | | | | | | | | | | | | | | |
|---|---|---|---|---|---|---|---|---|---|---|---|---|---|---|---|---|
| rs1667241 | upstream | *P-Value* | 0.95 | 0.21 | 0.058 | 0.053 | 0.088 | 0.61 | 0.77 | 0.85 | 0.22 | 0.78 | 0.91 | 0.85 | 0.39 | 0.5 |
| | | *Effect Size* | 0.021 | -0.29 | -0.6 | -1.2 | -0.6 | 0.15 | 0.085 | 0.046 | 0.34 | -0.068 | 0.026 | 0.11 | -0.28 | 0.4 |
| rs2704058 | upstream | *P-Value* | 0.19 | 0.59 | 0.27 | 0.54 | 0.67 | 0.23 | 0.37 | 0.45 | 0.78 | 0.45 | 0.52 | 0.7 | 0.41 | 0.8 |
| | | *Effect Size* | -0.12 | -0.041 | 0.11 | 0.065 | 0.031 | -0.093 | 0.09 | -0.041 | 0.022 | -0.06 | -0.048 | 0.039 | 0.077 | 0.04 |
| rs2704059 | upstream | *P-Value* | 0.98 | 0.32 | 0.058 | 0.053 | 0.088 | 0.41 | 0.77 | 0.84 | 0.22 | 0.97 | 0.91 | 0.85 | 0.51 | 0.5 |
| | | *Effect Size* | -0.0061 | -0.22 | -0.6 | -1.2 | -0.6 | 0.22 | 0.083 | 0.042 | 0.34 | -0.009 | 0.027 | 0.11 | -0.21 | 0.4 |
| rs9956836 | upstream | *P-Value* | 0.16 | 0.16 | 0.9 | 0.15 | 0.82 | 0.31 | 0.76 | 0.64 | 0.44 | 0.19 | 0.49 | 0.45 | 0.36 | 0.97 |
| | | *Effect Size* | 0.66 | 0.32 | 0.043 | -0.75 | -0.055 | -0.31 | -0.092 | -0.12 | -0.22 | -0.37 | -0.21 | 0.47 | 0.48 | 0.022 |
| rs1667242 | upstream | *P-Value* | 0.5 | 0.23 | 0.24 | 0.72 | 0.97 | 0.88 | 0.36 | 0.37 | 0.45 | 0.36 | 0.84 | 0.59 | 0.82 | 0.63 |
| | | *Effect Size* | -0.14 | -0.24 | -0.28 | -0.12 | 0.0067 | -0.03 | -0.2 | -0.12 | -0.14 | -0.17 | 0.037 | -0.14 | 0.06 | -0.17 |
| rs9956858 | upstream | *P-Value* | 0.092 | 0.14 | 0.77 | 0.14 | 0.91 | 0.33 | 0.81 | 0.63 | 0.39 | 0.23 | 0.48 | 0.45 | 0.32 | 0.97 |
| | | *Effect Size* | 0.79 | 0.34 | 0.11 | -0.79 | -0.028 | -0.29 | -0.072 | -0.12 | -0.25 | -0.34 | -0.21 | 0.47 | 0.52 | 0.025 |
| rs1791190 | upstream | *P-Value* | 0.28 | 0.27 | 0.17 | 0.54 | 1 | 0.48 | 0.8 | 0.88 | 0.54 | 0.54 | 0.27 | 0.2 | 0.87 | 0.42 |
| | | *Effect Size* | 0.25 | -0.17 | -0.32 | -0.22 | -0.00065 | 0.14 | 0.049 | -0.017 | 0.1 | -0.11 | 0.17 | 0.33 | 0.037 | 0.23 |
| rs75604963 | upstream | *P-Value* | 0.2 | 0.74 | 0.86 | 0.6 | 0.34 | 0.2 | 0.92 | 0.6 | 0.93 | 0.61 | 0.34 | 0.19 | 0.43 | 0.6 |
| | | *Effect Size* | 0.43 | -0.071 | -0.059 | 0.22 | 0.19 | 0.3 | 0.023 | -0.064 | -0.016 | -0.12 | 0.18 | 0.39 | 0.23 | 0.17 |
| rs1667243 | upstream | *P-Value* | 0.52 | 0.31 | 0.058 | 0.08 | 0.14 | 0.38 | 0.6 | 0.98 | 0.45 | 0.98 | 0.53 | 0.85 | 0.37 | 0.29 |
| | | *Effect Size* | 0.22 | -0.24 | -0.6 | -1.3 | -0.61 | 0.26 | 0.16 | 0.0075 | 0.22 | -0.0073 | -0.16 | 0.11 | -0.31 | 0.88 |
| rs11876866 | upstream | *P-Value* | 0.08 | 0.14 | 0.73 | 0.14 | 0.93 | 0.31 | 0.85 | 0.64 | 0.44 | 0.21 | 0.49 | 0.45 | 0.31 | 0.97 |
| | | *Effect Size* | 0.8 | 0.34 | 0.12 | -0.78 | -0.02 | -0.31 | -0.058 | -0.12 | -0.22 | -0.35 | -0.21 | 0.47 | 0.52 | 0.022 |
| rs1791188 | upstream | *P-Value* | 0.94 | 0.21 | 0.058 | 0.053 | 0.087 | 0.6 | 0.77 | 0.85 | 0.22 | 0.78 | 0.91 | 0.85 | 0.38 | 0.49 |
| | | *Effect Size* | 0.023 | -0.29 | -0.6 | -1.2 | -0.6 | 0.15 | 0.083 | 0.045 | 0.34 | -0.067 | 0.027 | 0.11 | -0.29 | 0.4 |
| rs6506932 | upstream | *P-Value* | 0.31 | 0.87 | 0.13 | 0.5 | 0.66 | 0.26 | 0.21 | 0.75 | 0.57 | 0.74 | 0.49 | 0.54 | 0.47 | 0.64 |
| | | *Effect Size* | -0.094 | -0.012 | 0.15 | 0.072 | 0.033 | -0.085 | 0.12 | -0.017 | 0.045 | -0.027 | -0.052 | 0.063 | 0.07 | 0.074 |
| rs7243588 | upstream | *P-Value* | 0.14 | 0.86 | 0.13 | 0.4 | 0.43 | 0.25 | 0.49 | 0.49 | 0.93 | 0.58 | 0.41 | 0.71 | 0.38 | 0.79 |
| | | *Effect Size* | -0.14 | -0.013 | 0.15 | 0.089 | 0.059 | -0.087 | 0.069 | -0.037 | -0.0064 | -0.044 | -0.063 | 0.039 | 0.083 | -0.044 |
| rs1616887 | upstream | *P-Value* | 0.19 | 0.6 | 0.71 | 0.66 | 0.71 | 0.49 | 0.47 | 0.6 | 0.8 | 0.57 | 0.31 | 0.6 | 0.86 | 0.88 |
| | | *Effect Size* | -0.12 | -0.038 | 0.037 | 0.047 | 0.028 | -0.054 | 0.074 | -0.027 | 0.02 | -0.045 | -0.078 | 0.053 | 0.017 | 0.025 |
| rs11662311 | upstream | *P-Value* | 0.081 | 0.58 | 0.17 | 0.41 | 0.92 | 0.33 | 0.19 | 0.96 | 0.4 | 0.82 | 0.31 | 0.99 | 0.84 | 0.82 |
| | | *Effect Size* | -0.16 | -0.042 | 0.14 | 0.092 | 0.0082 | -0.073 | 0.13 | -0.0031 | 0.068 | 0.018 | -0.079 | 0.0011 | 0.02 | 0.04 |
| rs4799580 | upstream | *P-Value* | 0.14 | 0.75 | 0.98 | 0.57 | 0.34 | 0.64 | 0.93 | 0.79 | 0.89 | 0.59 | 0.21 | 0.17 | 0.32 | 0.59 |
| | | *Effect Size* | 0.5 | -0.066 | 0.0081 | 0.23 | 0.19 | 0.11 | 0.02 | -0.033 | -0.028 | -0.13 | 0.25 | 0.41 | 0.3 | 0.17 |
| rs16962173 | upstream | *P-Value* | 0.071 | 0.14 | 0.67 | 0.15 | 0.97 | 0.31 | 0.88 | 0.64 | 0.44 | 0.23 | 0.49 | 0.45 | 0.31 | 0.97 |
| | | *Effect Size* | 0.79 | 0.34 | 0.15 | -0.75 | -0.0077 | -0.31 | -0.045 | -0.12 | -0.22 | -0.33 | -0.21 | 0.47 | 0.5 | 0.022 |

| | | | | | | | | | | | | | | | | |
|---|---|---|---|---|---|---|---|---|---|---|---|---|---|---|---|---|
| rs77507420 | upstream | *P-Value* | 0.48 | 0.76 | 0.12 | 0.76 | 0.91 | 0.51 | 0.44 | 0.41 | 0.91 | 0.52 | 0.11 | 0.96 | 0.48 | 0.78 |
| | | *Effect Size* | 0.2 | -0.078 | -0.49 | -0.14 | -0.036 | 0.19 | 0.28 | 0.13 | 0.034 | 0.18 | -0.47 | 0.023 | -0.27 | -0.12 |
| rs2420813 | upstream | *P-Value* | 0.033 | 0.55 | 0.18 | 0.32 | 0.73 | 0.3 | 0.49 | 0.71 | 0.82 | 0.94 | 0.35 | 0.82 | 0.53 | 0.85 |
| | | *Effect Size* | -0.18 | -0.043 | 0.13 | 0.11 | 0.026 | -0.076 | 0.067 | -0.02 | 0.017 | -0.0062 | -0.068 | -0.024 | 0.058 | -0.03 |
| rs118008746 | upstream | *P-Value* | 0.47 | 0.81 | 0.65 | 0.7 | 0.93 | 0.39 | 0.12 | 0.17 | 0.45 | 0.29 | 0.49 | 0.83 | 0.87 | 0.84 |
| | | *Effect Size* | -0.2 | 0.062 | 0.2 | 0.13 | 0.023 | 0.25 | -0.48 | 0.23 | -0.2 | -0.32 | 0.19 | 0.088 | -0.054 | 0.18 |
| rs4799581 | upstream | *P-Value* | 0.33 | 0.86 | 0.14 | 0.54 | 0.65 | 0.29 | 0.21 | 0.8 | 0.58 | 0.79 | 0.5 | 0.54 | 0.47 | 0.64 |
| | | *Effect Size* | -0.091 | -0.013 | 0.14 | 0.065 | 0.034 | -0.08 | 0.12 | -0.013 | 0.043 | -0.021 | -0.051 | 0.063 | 0.07 | 0.074 |
| rs8094790 | upstream | *P-Value* | 0.22 | 0.85 | 0.89 | 0.54 | 0.47 | 0.74 | 0.23 | 0.4 | 0.11 | 0.32 | 0.91 | 0.55 | 0.18 | 0.44 |
| | | *Effect Size* | -0.28 | -0.045 | 0.034 | 0.18 | 0.16 | -0.068 | -0.26 | -0.11 | -0.34 | -0.23 | 0.022 | -0.15 | 0.38 | -0.23 |
| rs1791187 | upstream | *P-Value* | 0.51 | 0.21 | 0.058 | 0.053 | 0.14 | 0.85 | 0.72 | 0.93 | 0.31 | 0.71 | 0.92 | 0.85 | 0.14 | 0.42 |
| | | *Effect Size* | 0.22 | -0.29 | -0.6 | -1.2 | -0.61 | 0.055 | 0.11 | -0.024 | 0.29 | -0.092 | 0.025 | 0.11 | -0.5 | 0.54 |
| rs1791186 | upstream | *P-Value* | 0.68 | 0.18 | 0.2 | 0.66 | 0.98 | 0.58 | 0.55 | 0.59 | 0.72 | 0.65 | 0.53 | 0.46 | 0.79 | 0.32 |
| | | *Effect Size* | -0.092 | -0.29 | -0.32 | 0.16 | 0.0065 | -0.12 | -0.12 | -0.086 | 0.072 | -0.097 | 0.12 | -0.21 | 0.07 | -0.42 |
| rs1791185 | upstream | *P-Value* | 0.018 | 0.95 | 0.35 | 0.22 | 0.84 | 0.89 | 0.86 | 0.65 | 0.98 | 0.19 | 0.52 | 0.14 | 0.8 | 0.46 |
| | | *Effect Size* | 0.53 | -0.0095 | -0.19 | -0.33 | 0.033 | -0.022 | 0.03 | -0.047 | 0.004 | -0.21 | 0.093 | 0.36 | 0.054 | 0.19 |
| rs1631020 | upstream | *P-Value* | 0.51 | 0.21 | 0.058 | 0.054 | 0.14 | 0.84 | 0.72 | 0.93 | 0.31 | 0.71 | 0.92 | 0.85 | 0.14 | 0.43 |
| | | *Effect Size* | 0.23 | -0.29 | -0.6 | -1.2 | -0.61 | 0.056 | 0.11 | -0.024 | 0.29 | -0.092 | 0.025 | 0.11 | -0.5 | 0.54 |
| rs79889942 | upstream | *P-Value* | 0.34 | 0.82 | 0.84 | 0.14 | 0.42 | 0.47 | 0.37 | 0.64 | 0.84 | 0.84 | 0.57 | 0.41 | 0.12 | 0.19 |
| | | *Effect Size* | -0.23 | -0.067 | 0.052 | 0.51 | 0.18 | -0.16 | -0.2 | -0.075 | -0.043 | -0.053 | 0.12 | -0.23 | 0.46 | -0.47 |
| rs140467514 | upstream | *P-Value* | 0.84 | 0.25 | 0.17 | 0.26 | 0.45 | 0.33 | 0.09 | 0.77 | 0.79 | 0.54 | 0.18 | 0.28 | 0.56 | 0.099 |
| | | *Effect Size* | -0.088 | -0.36 | 0.75 | 0.44 | 0.24 | -0.36 | 0.64 | -0.097 | 0.083 | -0.18 | -0.39 | 0.46 | 0.32 | 1.9 |
| rs16962179 | upstream | *P-Value* | 0.067 | 0.14 | 0.67 | 0.15 | 0.97 | 0.3 | 0.88 | 0.64 | 0.44 | 0.23 | 0.49 | 0.47 | 0.31 | 0.97 |
| | | *Effect Size* | 0.8 | 0.34 | 0.15 | -0.75 | -0.0083 | -0.31 | -0.043 | -0.12 | -0.22 | -0.33 | -0.21 | 0.45 | 0.5 | 0.029 |
| rs12962216 | upstream | *P-Value* | 0.67 | 0.25 | 0.42 | 0.3 | 0.22 | 0.93 | 0.88 | 0.41 | 1 | 0.44 | 0.71 | 0.63 | 0.42 | 0.24 |
| | | *Effect Size* | -0.06 | 0.13 | -0.13 | 0.17 | -0.15 | -0.01 | -0.024 | 0.063 | -0.00071 | -0.091 | -0.043 | -0.071 | -0.11 | -0.26 |
| rs61605601 | upstream | *P-Value* | 0.21 | 0.98 | 0.84 | 0.13 | 0.3 | 0.64 | 0.43 | 0.46 | 0.56 | 0.81 | 0.82 | 0.42 | 0.56 | 0.054 |
| | | *Effect Size* | -0.31 | -0.0071 | 0.052 | 0.52 | 0.22 | -0.1 | -0.17 | -0.12 | -0.13 | 0.062 | 0.048 | -0.23 | 0.17 | -0.63 |
| rs9989534 | upstream | *P-Value* | 0.15 | 0.7 | 0.038 | 0.067 | 0.28 | 0.25 | 0.11 | 0.51 | 0.94 | 0.6 | 0.11 | 0.62 | 0.33 | 0.34 |
| | | *Effect Size* | -0.62 | -0.16 | 1 | 1 | 0.35 | -0.42 | -0.6 | -0.14 | -0.029 | -0.19 | 0.6 | -0.24 | 0.48 | -0.54 |
| rs149397495 | upstream | *P-Value* | 0.025 | 0.3 | 0.75 | 0.44 | 0.63 | 0.91 | 0.87 | 0.98 | 0.74 | 0.41 | 0.41 | 0.94 | 0.12 | 0.58 |
| | | *Effect Size* | -0.9 | 0.34 | -0.16 | -0.38 | 0.25 | 0.041 | 0.08 | 0.005 | -0.14 | -0.27 | 0.28 | -0.031 | -0.93 | -0.62 |
| rs113035076 | upstream | *P-Value* | 0.62 | 0.074 | 0.92 | 0.27 | 0.65 | 0.12 | 0.67 | 0.93 | 0.5 | 0.76 | 0.91 | 0.69 | 0.51 | 0.72 |
| | | *Effect Size* | -0.17 | 0.46 | -0.045 | -0.54 | -0.17 | 0.45 | 0.15 | 0.017 | -0.18 | -0.076 | 0.04 | -0.17 | 0.21 | 0.26 |

| | | | | | | | | | | | | | | | | |
|---|---|---|---|---|---|---|---|---|---|---|---|---|---|---|---|---|
| rs2704061 | upstream | *P-Value* | 0.47 | 0.31 | 0.061 | 0.091 | 0.14 | 0.35 | 0.58 | 0.96 | 0.44 | 0.98 | 0.53 | 0.87 | 0.37 | 0.3 |
| | | *Effect Size* | 0.25 | -0.24 | -0.6 | -1.3 | -0.61 | 0.28 | 0.17 | 0.018 | 0.23 | -0.0052 | -0.16 | 0.1 | -0.31 | 0.89 |
| rs17740847 | upstream | *P-Value* | 0.35 | 0.44 | 0.4 | 0.45 | 0.15 | 0.9 | 0.26 | 0.13 | 0.55 | 0.26 | 0.045 | 0.66 | 0.057 | 0.17 |
| | | *Effect Size* | -0.12 | -0.086 | 0.13 | 0.12 | 0.17 | -0.015 | 0.16 | 0.13 | 0.071 | -0.14 | -0.23 | 0.069 | 0.26 | 0.35 |
| rs73418189 | upstream | *P-Value* | 0.15 | 0.7 | 0.038 | 0.067 | 0.28 | 0.25 | 0.11 | 0.51 | 0.94 | 0.6 | 0.11 | 0.62 | 0.33 | 0.34 |
| | | *Effect Size* | -0.62 | -0.16 | 1 | 1 | 0.35 | -0.42 | -0.6 | -0.14 | -0.029 | -0.19 | 0.6 | -0.24 | 0.48 | -0.54 |
| rs1574501 | upstream | *P-Value* | 0.14 | 0.75 | 0.94 | 0.57 | 0.33 | 0.64 | 0.94 | 0.79 | 0.88 | 0.6 | 0.2 | 0.17 | 0.32 | 0.6 |
| | | *Effect Size* | 0.5 | -0.066 | 0.026 | 0.23 | 0.19 | 0.11 | 0.018 | -0.033 | -0.029 | -0.12 | 0.25 | 0.41 | 0.3 | 0.17 |
| rs60939523 | upstream | *P-Value* | 0.38 | 0.79 | 0.87 | 0.13 | 0.26 | 0.35 | 0.46 | 0.48 | 0.46 | 0.93 | 0.46 | 0.41 | 0.28 | 0.22 |
| | | *Effect Size* | -0.21 | -0.08 | 0.042 | 0.52 | 0.25 | -0.2 | -0.17 | -0.11 | -0.16 | 0.022 | 0.15 | -0.23 | 0.33 | -0.42 |
| rs875120 | upstream | *P-Value* | 0.67 | 0.66 | 0.24 | 0.18 | 0.19 | 0.85 | 0.44 | 0.56 | 0.96 | 0.48 | 0.057 | 0.36 | 0.16 | 1 |
| | | *Effect Size* | 0.12 | -0.085 | 0.33 | 0.41 | 0.21 | -0.037 | -0.16 | -0.062 | -0.0084 | -0.13 | 0.33 | 0.24 | 0.37 | -0.0019 |
| rs875119 | upstream | *P-Value* | 0.14 | 0.75 | 0.98 | 0.57 | 0.34 | 0.64 | 0.93 | 0.79 | 0.89 | 0.59 | 0.21 | 0.17 | 0.32 | 0.59 |
| | | *Effect Size* | 0.5 | -0.066 | 0.0081 | 0.23 | 0.19 | 0.11 | 0.02 | -0.033 | -0.028 | -0.13 | 0.25 | 0.41 | 0.3 | 0.17 |
| rs76431866 | upstream | *P-Value* | 0.47 | 0.81 | 0.65 | 0.7 | 0.92 | 0.4 | 0.16 | 0.18 | 0.53 | 0.32 | 0.49 | 0.83 | 0.87 | 0.83 |
| | | *Effect Size* | -0.2 | 0.061 | 0.2 | 0.14 | 0.024 | 0.25 | -0.45 | 0.23 | -0.17 | -0.31 | 0.2 | 0.088 | -0.054 | 0.19 |
| rs1667244 | upstream | *P-Value* | 0.093 | 0.35 | 0.37 | 0.43 | 0.63 | 0.51 | 0.43 | 0.61 | 0.67 | 0.74 | 0.63 | 0.72 | 0.59 | 0.94 |
| | | *Effect Size* | -0.15 | -0.07 | 0.087 | 0.085 | 0.036 | -0.05 | 0.079 | -0.028 | 0.033 | -0.026 | -0.036 | 0.036 | 0.052 | -0.012 |
| rs1667245 | upstream | *P-Value* | 0.68 | 0.12 | 0.062 | 0.23 | 0.071 | 0.74 | 0.73 | 0.26 | 0.93 | 0.34 | 0.66 | 0.54 | 0.57 | 0.62 |
| | | *Effect Size* | -0.13 | -0.33 | -0.57 | -0.57 | -0.52 | 0.085 | -0.091 | 0.24 | 0.021 | -0.22 | 0.098 | -0.34 | -0.17 | 0.29 |
| rs76184052 | upstream | *P-Value* | 0.13 | 0.16 | 0.92 | 0.15 | 0.8 | 0.28 | 0.77 | 0.64 | 0.43 | 0.19 | 0.48 | 0.5 | 0.36 | 0.95 |
| | | *Effect Size* | 0.71 | 0.32 | 0.038 | -0.75 | -0.061 | -0.32 | -0.09 | -0.12 | -0.23 | -0.37 | -0.21 | 0.42 | 0.48 | 0.045 |
| rs72922938 | upstream | *P-Value* | 0.95 | 0.71 | 0.31 | 0.37 | 0.45 | 0.55 | 0.64 | 0.69 | 0.13 | 0.74 | 0.41 | 0.78 | 0.21 | 0.51 |
| | | *Effect Size* | 0.03 | -0.13 | -0.45 | 0.43 | 0.23 | -0.19 | -0.19 | 0.097 | 0.49 | -0.1 | -0.23 | -0.15 | 0.49 | 0.47 |
| rs111395060 | upstream | *P-Value* | 0.12 | 0.99 | 0.98 | 0.57 | 0.23 | 0.64 | 0.98 | 0.88 | 0.66 | 0.81 | 0.35 | 0.13 | 0.25 | 0.67 |
| | | *Effect Size* | 0.52 | -0.0031 | 0.0078 | 0.23 | 0.3 | 0.11 | -0.0051 | -0.021 | -0.096 | -0.057 | 0.2 | 0.45 | 0.34 | 0.17 |
| rs73418200 | upstream | *P-Value* | 0.68 | 0.77 | 0.76 | 0.17 | 0.27 | 0.37 | 0.78 | 0.093 | 0.94 | 0.51 | 0.41 | 0.41 | 0.32 | 0.15 |
| | | *Effect Size* | -0.1 | 0.099 | 0.081 | 0.45 | 0.25 | -0.21 | -0.067 | -0.31 | 0.02 | 0.21 | 0.19 | -0.23 | 0.34 | -0.6 |
| rs80322202 | upstream | *P-Value* | 0.32 | 0.97 | 0.94 | 0.17 | 0.89 | 0.51 | 0.32 | 0.36 | 0.018 | 0.08 | 0.32 | 0.72 | 0.59 | 0.51 |
| | | *Effect Size* | -0.56 | -0.018 | -0.034 | -0.84 | -0.074 | 0.28 | -0.72 | -0.24 | -0.94 | -0.61 | -0.45 | 0.23 | -0.54 | 0.42 |
| rs150555470 | upstream | *P-Value* | 0.2 | 0.6 | 0.55 | 0.61 | 0.028 | 0.53 | 0.36 | 0.8 | 0.7 | 0.47 | 0.023 | 0.36 | 0.46 | 0.37 |
| | | *Effect Size* | -0.62 | 0.17 | 0.26 | 0.2 | 0.8 | -0.19 | -0.33 | 0.064 | -0.12 | -0.28 | 0.63 | -0.36 | -0.3 | -1.2 |
| rs3764479 | upstream | *P-Value* | 0.081 | 0.56 | 0.21 | 0.55 | 0.93 | 0.42 | 0.22 | 0.92 | 0.28 | 0.92 | 0.51 | 0.97 | 0.84 | 0.71 |
| | | *Effect Size* | -0.16 | -0.044 | 0.13 | 0.068 | 0.0073 | -0.062 | 0.13 | -0.0053 | 0.089 | 0.0079 | -0.051 | 0.0036 | 0.02 | 0.064 |

| | | | | | | | | | | | | | | | | |
|---|---|---|---|---|---|---|---|---|---|---|---|---|---|---|---|---|
| rs13381522 | upstream | *P-Value* | 0.56 | 0.66 | 0.63 | 0.18 | 0.1 | 0.95 | 0.78 | 0.25 | 0.76 | 0.75 | 0.34 | 0.61 | 0.17 | 0.15 |
| | | *Effect Size* | 0.13 | 0.081 | 0.1 | 0.34 | 0.25 | 0.011 | -0.053 | -0.13 | -0.049 | 0.062 | 0.16 | 0.11 | 0.32 | -0.46 |
| rs1667246 | upstream | *P-Value* | 0.34 | 0.29 | 0.071 | 0.15 | 0.14 | 0.33 | 0.46 | 0.89 | 0.41 | 0.85 | 0.52 | 0.94 | 0.31 | 0.32 |
| | | *Effect Size* | 0.34 | -0.26 | -0.59 | -1.3 | -0.61 | 0.3 | 0.23 | 0.056 | 0.25 | -0.048 | -0.16 | 0.05 | -0.36 | 0.93 |
| rs3764478 | upstream | *P-Value* | 0.38 | 0.4 | 0.4 | 0.44 | 0.16 | 0.96 | 0.28 | 0.14 | 0.49 | 0.18 | 0.11 | 0.67 | 0.059 | 0.12 |
| | | *Effect Size* | -0.12 | -0.091 | 0.13 | 0.12 | 0.16 | -0.006 | 0.15 | 0.12 | 0.083 | -0.16 | -0.18 | 0.068 | 0.26 | 0.39 |
| rs72922940 | upstream | *P-Value* | 0.24 | 0.28 | 0.049 | 0.39 | 0.94 | 0.27 | 0.5 | 0.031 | 0.4 | 0.032 | 0.41 | 0.9 | 0.51 | 0.69 |
| | | *Effect Size* | -0.15 | -0.12 | 0.29 | -0.15 | -0.0093 | -0.13 | 0.095 | -0.16 | 0.097 | 0.25 | 0.085 | 0.016 | -0.091 | 0.11 |
| rs3764477 | upstream | *P-Value* | 0.12 | 0.99 | 0.98 | 0.58 | 0.23 | 0.63 | 0.95 | 0.88 | 0.62 | 0.86 | 0.35 | 0.13 | 0.24 | 0.67 |
| | | *Effect Size* | 0.52 | -0.0015 | 0.0071 | 0.23 | 0.3 | 0.12 | -0.016 | -0.021 | -0.11 | -0.042 | 0.21 | 0.45 | 0.34 | 0.17 |
| rs58616646 | upstream | *P-Value* | 0.66 | 0.67 | 0.63 | 0.18 | 0.14 | 0.83 | 0.66 | 0.34 | 0.99 | 0.81 | 0.43 | 0.61 | 0.08 | 0.12 |
| | | *Effect Size* | 0.1 | 0.078 | 0.1 | 0.34 | 0.24 | 0.037 | -0.084 | -0.11 | 0.0021 | 0.046 | 0.13 | 0.11 | 0.41 | -0.52 |
| rs116409170 | upstream | *P-Value* | 0.83 | 0.075 | 0.19 | 0.54 | 0.89 | 0.85 | 0.94 | 0.74 | 0.75 | 0.31 | 0.38 | 0.83 | 0.67 | 0.57 |
| | | *Effect Size* | 0.053 | -0.38 | 0.46 | -0.24 | -0.032 | -0.049 | 0.027 | 0.051 | -0.075 | 0.26 | 0.19 | -0.057 | 0.13 | 0.24 |
| rs79748512 | upstream | *P-Value* | 0.13 | 1 | 0.62 | 0.39 | 0.98 | 0.56 | 0.49 | 0.44 | 0.55 | 0.35 | 0.42 | 0.66 | 1 | 0.52 |
| | | *Effect Size* | 0.44 | 0.001 | -0.12 | -0.23 | -0.005 | 0.11 | -0.16 | -0.12 | 0.11 | 0.2 | 0.16 | -0.14 | -0.00071 | -0.44 |
| rs7231173 | intronic | *P-Value* | 0.18 | 0.72 | 0.035 | 0.077 | 0.28 | 0.27 | 0.12 | 0.51 | 0.99 | 0.6 | 0.1 | 0.62 | 0.34 | 0.34 |
| | | *Effect Size* | -0.57 | -0.15 | 1.1 | 0.99 | 0.36 | -0.4 | -0.59 | -0.14 | 0.0063 | -0.19 | 0.62 | -0.24 | 0.46 | -0.53 |
| rs723744 | intronic | *P-Value* | 0.058 | 0.25 | 0.31 | 0.45 | 0.94 | 0.29 | 0.47 | 0.97 | 0.41 | 0.8 | 0.76 | 0.96 | 0.95 | 0.84 |
| | | *Effect Size* | -0.16 | -0.085 | 0.097 | 0.084 | 0.006 | -0.077 | 0.069 | -0.0021 | 0.064 | -0.019 | -0.022 | -0.0057 | 0.0053 | 0.033 |
| rs73420308 | intronic | *P-Value* | 0.19 | 0.72 | 0.034 | 0.081 | 0.28 | 0.28 | 0.12 | 0.51 | 0.95 | 0.6 | 0.098 | 0.62 | 0.34 | 0.34 |
| | | *Effect Size* | -0.56 | -0.14 | 1.1 | 0.98 | 0.36 | -0.39 | -0.59 | -0.14 | 0.021 | -0.19 | 0.62 | -0.24 | 0.46 | -0.53 |
| rs1800458 | missense | *P-Value* | 0.9 | 0.23 | 0.4 | 0.27 | 0.96 | 0.18 | 0.31 | 0.51 | 0.4 | 0.59 | 0.72 | 0.21 | 0.57 | 0.79 |
| | | *Effect Size* | -0.018 | -0.15 | 0.17 | 0.23 | 0.0066 | -0.19 | -0.17 | 0.058 | -0.13 | 0.081 | 0.054 | 0.22 | -0.1 | -0.063 |
| rs1080093 | intronic | *P-Value* | 0.095 | 0.35 | 0.4 | 0.46 | 0.63 | 0.55 | 0.45 | 0.63 | 0.65 | 0.77 | 0.66 | 0.73 | 0.59 | 0.94 |
| | | *Effect Size* | -0.15 | -0.07 | 0.082 | 0.081 | 0.036 | -0.046 | 0.077 | -0.027 | 0.035 | -0.022 | -0.033 | 0.036 | 0.051 | -0.012 |
| rs72922947 | intronic | *P-Value* | 0.76 | 0.76 | 0.33 | 0.35 | 0.47 | 0.54 | 0.62 | 0.64 | 0.11 | 0.75 | 0.44 | 0.79 | 0.2 | 0.49 |
| | | *Effect Size* | 0.15 | -0.11 | -0.44 | 0.46 | 0.22 | -0.19 | -0.2 | 0.12 | 0.51 | -0.1 | -0.21 | -0.15 | 0.51 | 0.49 |
| rs1080094 | intronic | *P-Value* | 0.16 | 0.61 | 0.38 | 0.66 | 0.68 | 0.37 | 0.5 | 0.54 | 0.83 | 0.51 | 0.53 | 0.65 | 0.48 | 0.95 |
| | | *Effect Size* | -0.13 | -0.039 | 0.085 | 0.047 | 0.031 | -0.07 | 0.069 | -0.033 | 0.017 | -0.053 | -0.047 | 0.047 | 0.068 | -0.0096 |
| rs13381331 | intronic | *P-Value* | 0.22 | 0.74 | 0.033 | 0.092 | 0.28 | 0.3 | 0.12 | 0.51 | 0.88 | 0.6 | 0.093 | 0.62 | 0.34 | 0.34 |
| | | *Effect Size* | -0.52 | -0.13 | 1.1 | 0.94 | 0.36 | -0.37 | -0.58 | -0.14 | 0.058 | -0.19 | 0.63 | -0.24 | 0.46 | -0.53 |
| rs59882235 | intronic | *P-Value* | 0.72 | 0.55 | 0.38 | 0.81 | 0.61 | 0.71 | 0.8 | 0.15 | 0.45 | 0.95 | 0.39 | 0.61 | 0.79 | 0.72 |
| | | *Effect Size* | 0.091 | 0.21 | -0.26 | -0.086 | 0.19 | 0.091 | 0.068 | -0.26 | -0.19 | -0.021 | -0.21 | -0.17 | 0.11 | -0.18 |

| | | | | | | | | | | | | | | | | |
|---|---|---|---|---|---|---|---|---|---|---|---|---|---|---|---|---|
| rs75075253 | intronic | *P-Value* | 0.81 | 0.42 | 0.36 | 0.76 | 0.92 | 0.94 | 0.53 | 0.39 | 0.69 | 0.18 | 0.67 | 0.5 | 0.27 | 0.29 |
| | | *Effect Size* | 0.064 | 0.37 | -0.29 | 0.12 | 0.042 | 0.019 | 0.18 | -0.18 | 0.11 | 0.56 | -0.11 | -0.23 | 0.48 | -0.64 |
| rs3764476 | intronic | *P-Value* | 0.06 | 0.59 | 0.4 | 0.76 | 0.93 | 0.26 | 0.27 | 0.58 | 0.57 | 0.87 | 0.37 | 0.94 | 0.76 | 0.82 |
| | | *Effect Size* | -0.17 | -0.041 | 0.081 | 0.034 | 0.0066 | -0.084 | 0.11 | -0.03 | 0.045 | 0.013 | -0.068 | -0.0076 | 0.03 | 0.038 |
| rs7235277 | intronic | *P-Value* | 0.066 | 0.6 | 0.48 | 0.66 | 0.99 | 0.29 | 0.2 | 0.64 | 0.35 | 0.79 | 0.5 | 0.91 | 0.73 | 0.91 |
| | | *Effect Size* | -0.16 | -0.04 | 0.069 | 0.049 | -0.00089 | -0.079 | 0.13 | -0.025 | 0.073 | 0.021 | -0.051 | -0.012 | 0.033 | 0.018 |
| rs3794884 | intronic | *P-Value* | 0.067 | 0.6 | 0.5 | 0.83 | 0.95 | 0.25 | 0.21 | 0.57 | 0.58 | 1 | 0.41 | 0.95 | 0.72 | 0.82 |
| | | *Effect Size* | -0.16 | -0.04 | 0.065 | 0.023 | -0.005 | -0.087 | 0.13 | -0.031 | 0.044 | 0.00012 | -0.063 | -0.006 | 0.034 | 0.039 |
| rs1667250 | intronic | *P-Value* | 0.25 | 0.32 | 0.42 | 0.37 | 0.37 | 0.068 | 0.51 | 0.4 | 0.19 | 0.73 | 0.93 | 0.58 | 0.26 | 0.44 |
| | | *Effect Size* | 0.42 | -0.24 | -0.28 | 1.4 | -0.45 | 0.61 | 0.21 | 0.36 | 0.46 | 0.088 | 0.023 | -0.39 | -0.38 | 0.79 |
| rs1791227 | intronic | *P-Value* | 0.77 | 0.7 | 0.71 | 0.093 | 0.8 | 0.69 | 0.55 | 0.24 | 0.73 | 0.25 | 0.51 | 0.6 | 0.72 | 0.66 |
| | | *Effect Size* | -0.068 | -0.084 | 0.1 | 0.69 | -0.077 | 0.094 | -0.12 | 0.19 | 0.08 | 0.22 | -0.13 | -0.24 | -0.094 | -0.21 |
| rs1667251 | intronic | *P-Value* | 0.88 | 0.57 | 0.71 | 0.094 | 0.8 | 0.74 | 0.66 | 0.24 | 0.78 | 0.31 | 0.51 | 0.6 | 0.54 | 0.67 |
| | | *Effect Size* | -0.036 | -0.12 | 0.1 | 0.69 | -0.077 | 0.079 | -0.091 | 0.18 | 0.063 | 0.2 | -0.13 | -0.23 | -0.16 | -0.21 |
| rs36204272 | intronic | *P-Value* | 0.6 | 0.7 | 0.33 | 0.31 | 0.14 | 0.44 | 0.61 | 0.51 | 0.86 | 0.33 | 0.043 | 0.28 | 0.35 | 0.67 |
| | | *Effect Size* | 0.15 | -0.083 | 0.29 | 0.33 | 0.27 | 0.16 | -0.12 | -0.079 | -0.035 | -0.2 | 0.4 | 0.3 | 0.25 | -0.16 |
| rs62093482 | downstream | *P-Value* | 0.27 | 0.55 | 0.29 | 0.23 | 0.46 | 0.063 | 0.35 | 0.4 | 0.62 | 0.096 | 0.95 | 0.88 | 0.41 | 0.081 |
| | | *Effect Size* | -0.33 | 0.16 | -0.37 | 0.4 | -0.23 | 0.51 | -0.31 | -0.16 | -0.15 | -0.42 | -0.016 | 0.068 | -0.23 | 0.93 |
| rs1791228 | downstream | *P-Value* | 0.17 | 0.23 | 0.22 | 0.41 | 0.62 | 0.093 | 0.71 | 0.9 | 0.8 | 0.88 | 0.62 | 0.5 | 0.86 | 0.54 |
| | | *Effect Size* | -0.12 | -0.091 | 0.12 | 0.087 | 0.039 | -0.13 | -0.036 | 0.0064 | -0.019 | 0.012 | -0.036 | 0.074 | 0.016 | -0.1 |
| rs75032823 | downstream | *P-Value* | 0.21 | 0.45 | 0.42 | 0.37 | 0.16 | 0.99 | 0.3 | 0.18 | 0.6 | 0.24 | 0.17 | 0.66 | 0.083 | 0.11 |
| | | *Effect Size* | -0.16 | -0.082 | 0.12 | 0.14 | 0.16 | -0.0014 | 0.14 | 0.11 | 0.061 | -0.14 | -0.15 | 0.069 | 0.24 | 0.4 |
| rs77973165 | downstream | *P-Value* | 0.45 | 0.38 | 0.15 | 0.57 | 0.52 | 0.55 | 0.46 | 0.15 | 0.12 | 0.95 | 0.29 | 0.69 | 0.52 | 0.56 |
| | | *Effect Size* | 0.21 | 0.35 | -0.44 | -0.22 | -0.22 | -0.15 | 0.21 | -0.29 | -0.39 | 0.022 | -0.26 | -0.14 | 0.3 | -0.34 |
| rs113289164 | downstream | *P-Value* | 0.85 | 0.59 | 0.8 | 0.82 | 0.22 | 0.57 | 0.74 | 0.7 | 0.32 | 0.34 | 0.21 | 0.26 | 0.65 | 0.81 |
| | | *Effect Size* | -0.051 | 0.1 | -0.072 | -0.074 | 0.28 | 0.12 | -0.075 | -0.048 | -0.19 | -0.19 | 0.23 | 0.28 | 0.12 | 0.093 |
| rs117207032 | downstream | *P-Value* | 0.36 | 0.3 | 0.17 | 0.046 | 0.82 | 0.41 | 0.63 | 0.19 | 0.31 | 0.93 | 0.74 | 0.77 | 0.27 | 0.38 |
| | | *Effect Size* | -0.47 | 0.44 | 0.75 | 1.4 | -0.085 | -0.31 | -0.25 | -0.39 | -0.4 | -0.034 | 0.12 | -0.14 | -0.46 | -0.71 |
| rs1791229 | downstream | *P-Value* | 0.096 | 0.38 | 0.4 | 0.46 | 0.73 | 0.55 | 0.52 | 0.65 | 0.76 | 0.76 | 0.75 | 0.81 | 0.58 | 0.93 |
| | | *Effect Size* | -0.15 | -0.065 | 0.082 | 0.081 | 0.026 | -0.047 | 0.065 | -0.025 | 0.024 | -0.024 | -0.024 | 0.025 | 0.053 | -0.015 |
| rs1667252 | downstream | *P-Value* | 0.73 | 0.44 | 0.91 | 0.088 | 0.79 | 0.14 | 0.96 | 0.15 | 0.36 | 0.68 | 0.68 | 0.49 | 0.24 | 0.48 |
| | | *Effect Size* | -0.081 | -0.17 | 0.029 | 1.1 | -0.083 | 0.4 | 0.011 | 0.36 | 0.24 | 0.087 | -0.082 | -0.33 | -0.31 | -0.38 |
| rs4799583 | downstream | *P-Value* | 0.16 | 0.58 | 0.44 | 0.78 | 0.69 | 0.5 | 0.26 | 0.28 | 0.67 | 0.53 | 0.96 | 0.72 | 0.47 | 0.96 |
| | | *Effect Size* | -0.13 | -0.042 | 0.077 | 0.03 | 0.031 | -0.052 | 0.11 | -0.058 | 0.034 | -0.05 | -0.0037 | 0.037 | 0.068 | 0.0084 |

| | | | | | | | | | | | | | | | | |
|---|---|---|---|---|---|---|---|---|---|---|---|---|---|---|---|---|
| rs1473342 | downstream | *P-Value* | 0.062 | 0.55 | 0.35 | 0.91 | 0.93 | 0.43 | 0.26 | 0.68 | 0.27 | 0.79 | 0.61 | 0.94 | 0.79 | 0.67 |
| | | *Effect Size* | -0.17 | -0.046 | 0.097 | 0.013 | -0.007 | -0.061 | 0.12 | -0.022 | 0.092 | -0.022 | -0.04 | 0.0077 | 0.026 | 0.073 |
| rs1900880 | downstream | *P-Value* | 0.6 | 0.7 | 0.33 | 0.31 | 0.14 | 0.44 | 0.59 | 0.51 | 0.84 | 0.32 | 0.043 | 0.28 | 0.35 | 0.67 |
| | | *Effect Size* | 0.15 | -0.083 | 0.29 | 0.33 | 0.27 | 0.16 | -0.12 | -0.079 | -0.039 | -0.2 | 0.4 | 0.3 | 0.25 | -0.16 |
| rs1791201 | downstream | *P-Value* | 0.16 | 0.47 | 0.61 | 0.57 | 0.94 | 0.5 | 0.51 | 0.84 | 0.94 | 0.94 | 0.46 | 0.72 | 0.55 | 0.78 |
| | | *Effect Size* | -0.13 | -0.054 | 0.051 | 0.062 | 0.0058 | -0.052 | 0.065 | -0.011 | 0.0061 | -0.0058 | -0.055 | 0.036 | 0.058 | 0.045 |
| rs1667253 | downstream | *P-Value* | 0.76 | 0.48 | 0.88 | 0.058 | 0.53 | 0.13 | 0.91 | 0.17 | 0.37 | 0.73 | 0.87 | 0.49 | 0.53 | 0.57 |
| | | *Effect Size* | -0.074 | -0.16 | -0.042 | 1.3 | -0.24 | 0.41 | -0.026 | 0.32 | 0.24 | 0.075 | 0.035 | -0.33 | -0.17 | 0.35 |
| rs9962355 | downstream | *P-Value* | 0.085 | 0.17 | 0.94 | 0.15 | 0.76 | 0.19 | 0.78 | 0.64 | 0.39 | 0.17 | 0.41 | 0.62 | 0.36 | 0.9 |
| | | *Effect Size* | 0.8 | 0.31 | 0.027 | -0.75 | -0.074 | -0.4 | -0.086 | -0.12 | -0.25 | -0.38 | -0.25 | 0.31 | 0.48 | 0.089 |
| rs1667254 | downstream | *P-Value* | 0.1 | 0.43 | 0.42 | 0.68 | 0.9 | 0.71 | 0.34 | 0.96 | 0.2 | 0.9 | 0.67 | 0.93 | 0.93 | 0.56 |
| | | *Effect Size* | -0.14 | -0.059 | 0.077 | 0.046 | -0.01 | -0.028 | 0.096 | -0.0025 | 0.1 | -0.0095 | -0.031 | -0.0095 | -0.0084 | 0.097 |
| rs148278972 | downstream | *P-Value* | 0.77 | 0.17 | 0.89 | 0.24 | 0.081 | 0.4 | 0.56 | 0.54 | 0.0083 | 0.4 | 0.24 | 0.48 | 0.32 | 0.58 |
| | | *Effect Size* | -0.083 | -0.36 | -0.058 | 0.52 | -0.61 | -0.33 | 0.22 | 0.14 | -1 | 0.36 | -0.38 | -0.26 | 0.35 | 0.37 |
| rs17740990 | downstream | *P-Value* | 0.12 | 0.98 | 0.99 | 0.59 | 0.23 | 0.55 | 0.66 | 0.88 | 0.43 | 0.73 | 0.33 | 0.13 | 0.24 | 0.66 |
| | | *Effect Size* | 0.51 | 0.0064 | 0.0029 | 0.23 | 0.3 | 0.14 | -0.11 | -0.02 | -0.16 | -0.08 | 0.21 | 0.45 | 0.34 | 0.17 |
| rs1611949 | downstream | *P-Value* | 0.09 | 0.32 | 0.2 | 0.36 | 0.56 | 0.66 | 0.78 | 0.94 | 0.51 | 0.66 | 0.92 | 0.72 | 0.76 | 0.93 |
| | | *Effect Size* | -0.15 | -0.074 | 0.12 | 0.099 | 0.046 | -0.033 | 0.027 | -0.0037 | 0.05 | -0.034 | 0.0075 | 0.037 | 0.028 | 0.014 |
| rs112749152 | downstream | *P-Value* | 0.12 | 0.96 | 0.98 | 0.57 | 0.23 | 0.6 | 0.67 | 0.88 | 0.44 | 0.63 | 0.38 | 0.14 | 0.26 | 0.67 |
| | | *Effect Size* | 0.52 | -0.012 | 0.0068 | 0.23 | 0.3 | 0.13 | -0.11 | -0.022 | -0.16 | -0.11 | 0.19 | 0.44 | 0.33 | 0.17 |
| rs1667255 | downstream | *P-Value* | 0.13 | 0.4 | 0.48 | 0.45 | 0.95 | 0.98 | 0.54 | 0.83 | 0.92 | 0.77 | 0.65 | 0.72 | 0.46 | 0.94 |
| | | *Effect Size* | -0.13 | -0.063 | 0.071 | 0.082 | 0.0053 | 0.0017 | 0.062 | -0.012 | 0.0078 | -0.022 | -0.034 | 0.037 | 0.07 | -0.012 |
| rs1791200 | downstream | *P-Value* | 0.078 | 0.35 | 0.34 | 0.51 | 0.75 | 0.74 | 0.62 | 0.73 | 0.54 | 0.86 | 0.31 | 0.9 | 0.8 | 0.79 |
| | | *Effect Size* | -0.15 | -0.069 | 0.091 | 0.073 | -0.026 | -0.024 | 0.047 | 0.018 | 0.048 | -0.013 | -0.075 | 0.013 | 0.023 | 0.045 |
| rs1791199 | downstream | *P-Value* | 0.13 | 0.38 | 0.39 | 0.45 | 0.95 | 0.93 | 0.58 | 0.79 | 0.99 | 0.62 | 0.71 | 0.72 | 0.44 | 0.94 |
| | | *Effect Size* | -0.14 | -0.065 | 0.086 | 0.084 | 0.0053 | -0.0066 | 0.057 | -0.015 | 0.00068 | -0.039 | -0.028 | 0.037 | 0.074 | -0.012 |
| rs1791198 | downstream | *P-Value* | 0.13 | 0.39 | 0.38 | 0.33 | 0.93 | 0.99 | 0.55 | 0.86 | 0.6 | 0.79 | 0.81 | 0.77 | 0.44 | 0.85 |
| | | *Effect Size* | -0.14 | -0.065 | 0.089 | 0.11 | 0.0074 | -0.00099 | 0.061 | -0.0093 | 0.041 | -0.021 | -0.018 | 0.03 | 0.074 | -0.031 |
| rs58114875 | downstream | *P-Value* | 0.55 | 0.65 | 0.59 | 0.064 | 0.52 | 0.36 | 0.56 | 0.17 | 0.72 | 0.89 | 0.92 | 0.49 | 0.51 | 0.58 |
| | | *Effect Size* | -0.17 | 0.17 | 0.17 | 1.3 | -0.25 | 0.28 | -0.18 | 0.31 | 0.11 | 0.045 | -0.031 | -0.33 | -0.28 | 0.34 |
| rs1667257 | downstream | *P-Value* | 0.081 | 0.53 | 0.38 | 0.58 | 0.72 | 0.72 | 0.44 | 0.63 | 0.52 | 0.77 | 0.54 | 0.84 | 0.59 | 0.98 |
| | | *Effect Size* | -0.15 | -0.047 | 0.088 | 0.062 | -0.028 | -0.027 | 0.077 | -0.025 | 0.051 | -0.023 | -0.046 | -0.021 | 0.051 | -0.0047 |
| rs77473169 | downstream | *P-Value* | 0.91 | 0.57 | 0.77 | 0.81 | 0.68 | 0.86 | 0.25 | 0.19 | 0.48 | 0.64 | 0.27 | NA | 0.78 | 0.4 |
| | | *Effect Size* | -0.048 | -0.21 | 0.19 | 0.14 | 0.23 | 0.068 | -0.51 | 0.36 | -0.25 | -0.18 | -0.44 | NA | 0.13 | -0.73 |

| | | | | | | | | | | | | | | | | |
|---|---|---|---|---|---|---|---|---|---|---|---|---|---|---|---|---|
| rs72922962 | downstream | *P-Value* | 0.78 | 0.76 | 0.33 | 0.36 | 0.47 | 0.53 | 0.62 | 0.64 | 0.11 | 0.74 | 0.44 | 0.78 | 0.2 | 0.49 |
| | | *Effect Size* | 0.14 | -0.11 | -0.44 | 0.45 | 0.22 | -0.2 | -0.2 | 0.12 | 0.52 | -0.1 | -0.21 | -0.15 | 0.5 | 0.49 |
| rs1667258 | downstream | *P-Value* | 0.2 | 0.52 | 0.49 | 0.45 | 0.87 | 0.87 | 0.56 | 0.97 | 0.78 | 0.94 | 0.46 | 0.68 | 0.4 | 0.86 |
| | | *Effect Size* | -0.11 | -0.048 | 0.071 | 0.085 | -0.014 | -0.012 | 0.058 | 0.0021 | 0.022 | 0.0057 | -0.056 | 0.043 | 0.081 | 0.028 |
| rs11664321 | downstream | *P-Value* | 0.098 | 0.7 | 0.12 | 0.37 | 0.57 | 0.36 | 0.62 | 0.62 | 0.99 | 0.93 | 0.31 | 0.78 | 0.81 | 0.67 |
| | | *Effect Size* | -0.14 | -0.029 | 0.15 | 0.1 | -0.048 | -0.068 | -0.048 | 0.026 | 0.0015 | -0.0063 | -0.077 | 0.03 | 0.022 | 0.068 |
| rs1791197 | downstream | *P-Value* | 0.16 | 0.51 | 0.25 | 0.39 | 0.87 | 0.71 | 0.97 | 0.86 | 0.8 | 0.86 | 0.75 | 0.76 | 0.47 | 0.67 |
| | | *Effect Size* | -0.13 | -0.05 | 0.12 | 0.097 | 0.013 | -0.028 | 0.004 | 0.0095 | 0.019 | -0.013 | -0.024 | 0.032 | 0.069 | -0.07 |
| rs57844152 | downstream | *P-Value* | 0.62 | 0.63 | 0.54 | 0.14 | 0.41 | 0.96 | 0.15 | 0.51 | 0.36 | 0.91 | 0.54 | 0.3 | 0.3 | 0.45 |
| | | *Effect Size* | 0.11 | 0.091 | 0.14 | 0.43 | 0.19 | -0.0086 | -0.26 | 0.071 | -0.15 | 0.02 | 0.1 | 0.26 | 0.23 | 0.25 |
| rs117591375 | downstream | *P-Value* | 0.92 | 0.54 | 0.26 | 0.27 | 0.83 | 0.29 | 0.35 | 0.62 | 0.23 | 0.37 | 0.021 | 0.63 | 0.83 | 0.84 |
| | | *Effect Size* | -0.068 | 0.37 | -0.4 | -0.44 | 0.32 | 0.48 | -0.35 | -0.21 | 0.45 | -0.28 | -0.65 | -0.31 | -0.078 | -0.17 |
| rs1791196 | downstream | *P-Value* | 0.68 | 0.87 | 0.96 | 0.088 | 0.39 | 0.75 | 0.47 | 0.65 | 0.98 | 0.84 | 0.12 | 0.82 | 0.32 | 0.67 |
| | | *Effect Size* | 0.081 | 0.028 | 0.0092 | 0.39 | 0.14 | 0.051 | -0.13 | -0.048 | -0.0035 | 0.032 | 0.23 | 0.05 | 0.21 | -0.14 |
| rs145583622 | downstream | *P-Value* | 0.46 | 0.063 | 0.25 | 0.13 | 0.58 | 0.57 | 0.58 | 0.17 | 0.69 | 0.061 | 0.42 | 0.89 | 0.81 | 0.65 |
| | | *Effect Size* | -0.28 | -0.64 | 0.49 | -1 | -0.22 | 0.2 | -0.27 | 0.39 | -0.14 | 0.64 | 0.26 | -0.062 | 0.13 | -0.31 |
| rs4799585 | downstream | *P-Value* | 0.29 | 0.69 | 0.47 | 0.84 | 0.59 | 0.72 | 0.69 | 0.96 | 0.45 | 0.95 | 0.91 | 0.59 | 0.77 | 0.74 |
| | | *Effect Size* | -0.1 | -0.031 | 0.08 | -0.024 | -0.048 | -0.029 | -0.042 | -0.003 | 0.064 | 0.0048 | -0.0091 | -0.062 | 0.03 | -0.057 |
| rs9951917 | downstream | *P-Value* | 0.049 | 0.19 | 0.98 | 0.15 | 0.71 | 0.13 | 0.8 | 0.64 | 0.35 | 0.17 | 0.37 | 0.79 | 0.37 | 0.84 |
| | | *Effect Size* | 0.88 | 0.3 | 0.01 | -0.75 | -0.091 | -0.46 | -0.078 | -0.12 | -0.28 | -0.39 | -0.28 | 0.15 | 0.48 | 0.13 |
| rs12458967 | downstream | *P-Value* | 0.13 | 0.98 | 0.82 | 0.7 | 0.22 | 0.52 | 0.61 | 0.75 | 0.4 | 0.6 | 0.4 | 0.12 | 0.29 | 0.53 |
| | | *Effect Size* | 0.5 | -0.0065 | 0.073 | 0.16 | 0.3 | 0.16 | -0.13 | -0.044 | -0.17 | -0.12 | 0.18 | 0.45 | 0.31 | 0.24 |
| rs4799586 | downstream | *P-Value* | 0.13 | 0.95 | 0.78 | 0.71 | 0.22 | 0.52 | 0.64 | 0.72 | 0.4 | 0.64 | 0.44 | 0.13 | 0.3 | 0.51 |
| | | *Effect Size* | 0.5 | -0.013 | 0.087 | 0.15 | 0.31 | 0.16 | -0.12 | -0.048 | -0.18 | -0.11 | 0.17 | 0.44 | 0.31 | 0.25 |
| rs9947375 | downstream | *P-Value* | 0.038 | 0.2 | 1 | 0.15 | 0.69 | 0.11 | 0.81 | 0.64 | 0.34 | 0.16 | 0.35 | 0.88 | 0.37 | 0.82 |
| | | *Effect Size* | 0.9 | 0.29 | 0.0015 | -0.75 | -0.099 | -0.49 | -0.074 | -0.12 | -0.29 | -0.39 | -0.29 | 0.082 | 0.48 | 0.14 |
| rs4799587 | downstream | *P-Value* | 0.13 | 0.95 | 0.77 | 0.72 | 0.22 | 0.52 | 0.65 | 0.71 | 0.4 | 0.65 | 0.45 | 0.13 | 0.3 | 0.51 |
| | | *Effect Size* | 0.5 | -0.014 | 0.091 | 0.14 | 0.31 | 0.16 | -0.12 | -0.05 | -0.18 | -0.11 | 0.17 | 0.44 | 0.3 | 0.25 |
| rs2595361 | downstream | *P-Value* | 0.48 | 0.86 | 0.22 | 0.27 | 0.56 | 0.76 | 0.12 | 0.53 | 0.17 | 0.029 | 0.29 | 0.079 | 0.94 | 0.59 |
| | | *Effect Size* | 0.087 | -0.019 | 0.18 | 0.19 | 0.075 | -0.033 | -0.18 | 0.045 | -0.15 | 0.24 | 0.11 | 0.26 | -0.01 | 0.11 |
| rs7232056 | downstream | *P-Value* | 0.45 | 0.41 | 0.68 | 0.31 | 0.55 | 0.83 | 0.67 | 0.53 | 0.43 | 0.66 | 0.95 | 0.13 | 0.79 | 0.65 |
| | | *Effect Size* | -0.07 | -0.063 | 0.043 | -0.11 | -0.047 | -0.017 | -0.042 | -0.035 | 0.063 | -0.035 | -0.0053 | -0.16 | -0.025 | -0.074 |
| rs11663991 | downstream | *P-Value* | 1 | 0.35 | 0.34 | 0.78 | 0.83 | 0.68 | 0.071 | 0.94 | 0.88 | 0.29 | 0.34 | 0.77 | 0.74 | 0.9 |
| | | *Effect Size* | 0.00029 | -0.069 | 0.092 | -0.03 | -0.019 | -0.03 | -0.16 | -0.0038 | -0.011 | 0.081 | 0.069 | -0.03 | -0.03 | 0.018 |

| | | | | | | | | | | | | | | | | |
|---|---|---|---|---|---|---|---|---|---|---|---|---|---|---|---|---|
| rs9797439 | downstream | *P-Value* | 0.14 | 0.93 | 0.77 | 0.74 | 0.22 | 0.52 | 0.67 | 0.68 | 0.39 | 0.67 | 0.49 | 0.13 | 0.32 | 0.5 |
| | | *Effect Size* | 0.5 | -0.02 | 0.092 | 0.13 | 0.31 | 0.15 | -0.11 | -0.055 | -0.18 | -0.1 | 0.15 | 0.42 | 0.3 | 0.26 |
| rs56861741 | downstream | *P-Value* | 0.94 | 0.78 | 0.29 | 0.27 | 0.89 | 0.22 | 0.15 | 0.34 | 0.33 | 0.0042 | 0.45 | 0.33 | 0.44 | 0.87 |
| | | *Effect Size* | -0.0092 | -0.034 | 0.16 | 0.2 | -0.018 | -0.14 | -0.19 | 0.074 | -0.12 | 0.36 | 0.087 | 0.16 | -0.12 | 0.034 |
| rs9962358 | downstream | *P-Value* | 0.63 | 0.93 | 0.15 | 0.84 | 0.34 | 0.94 | 0.56 | 0.87 | 0.43 | 0.55 | 0.46 | 0.54 | 0.25 | 0.3 |
| | | *Effect Size* | -0.14 | 0.033 | -0.42 | 0.08 | -0.32 | 0.019 | 0.16 | -0.032 | -0.19 | 0.19 | -0.18 | -0.21 | 0.48 | -0.56 |
