# Supplementary material for: Population diversity of the genetically determined TTR expression in human tissues and its implications in TTR amyloidosis
Source: BMC Genomics. 2017 Mar 23;18:254. doi: 10.1186/s12864-017-3646-1 (PMC5364715; doi:10.1186/s12864-017-3646-1)
Supplement: Supplementary file 10 — A) Clumped datasets for the African ancestry. Colors refer to variants in the LD blocks (r2 > 0.5; different colors represent different LD blocks) and X = presence of a variant in a specific dataset after the LD clumping analysis. B) Clumped datasets for the European ancestry. Color refer to variants in the LD block (r2 > 0.5; different colors represent different LD blocks) and X = presence of a variant in a specific dataset after the LD clumping analysis. C) Clumped datasets for the Eastern Asian ancestry. Colors refer to variants in the LD blocks (r2 > 0.5; different colors represent different LD blocks) and X = presence of a variant in a specific dataset after the LD clumping analysis D) Clumped datasets for the Southern Asian ancestry. Colors refer to variants in the LD blocks (r2 > 0.5; different colors represent different LD blocks) and X = presence of a variant in a specific dataset after the LD clumping analysis. E) Clumped datasets for the American ancestry. Colors refer to variants in the LD blocks (r2 > 0.5; different colors represent different LD blocks) and X = presence of a variant in a specific dataset after the LD clumping analysis. (PDF 88 kb) [file 12864_2017_3646_MOESM10_ESM.pdf]

Additional Data 11:

A) Clumped datasets for the African ancestry. Colors refer to variants in the LD blocks ($r^2 > 0.5$; different colors represent different LD blocks) and X = presence of a variant in a specific dataset after the LD clumping analysis.

RSID	Colon - Transverse	Colon - Sigmoid	Esophagus - Muscularis	Esophagus - Mucosa	Heart - Atrial Appendage	Heart - Left Ventricle	Liver	Muscle - Skeletal	Nerve - Tibial	Stomach	Small Intestine - Terminal	Adipose - Subcutaneous	Cells - Transformed fibroblasts	Skin - Sun Exposed (Lower leg)
rs8090089														
rs4799579														
rs17660933														
rs11081702														
rs1667238														
rs2034404	X													
rs1667239														
rs1375445	X													
rs1791191														
rs1375446														
rs1667241					X	X								
rs2704058														
rs2704059														
rs9956836														

rs1667242														
rs9956858														
rs1791190														
rs75604963														
rs1667243														
rs11876866														
rs1791188							X							
rs6506932														
rs7243588														
rs1616887														
rs11662311														
rs4799580														
rs16962173														
rs77507420														
rs2420813	X													
rs118008746														
rs4799581														
rs8094790														
rs1791187														
rs1791186														
rs1791185	X													
rs1631020														
rs79889942														
rs140467514									X		X			
rs16962179														
rs12962216														
rs61605601											X			
rs9989534						X								
rs149397495	X													
rs113035076			X											

rs2704061														
rs17740847				X										X
rs73418189														
rs1574501														
rs60939523														
rs875120														X
rs875119														
rs76431866														
rs1667244														
rs1667245					X		X							
rs76184052														
rs72922938														
rs111395060														
rs73418200										X				
rs80322202												X	X	
rs150555470							X							X
rs3764479														
rs13381522														
rs1667246														
rs3764478				X										
rs72922940					X					X			X	
rs3764477														
rs58616646				X										
rs116409170			X											
rs79748512														
rs7231173														
rs723744	X													
rs73420308														
rs1800458														
rs1080093														

rs72922947														
rs1080094														
rs13381331					X									X
rs59882235														
rs75075253														
rs3764476	X													
rs7235277														
rs3794884														
rs1667250								X						
rs1791227														
rs1667251														
rs36204272														X
rs62093482								X			X		X	
rs1791228								X						
rs75032823														
rs77973165														
rs113289164														
rs117207032						X								
rs1791229														
rs1667252														
rs4799583														
rs1473342	X													
rs1900880														
rs1791201														
rs1667253						X								
rs9962355														
rs1667254														
rs148278972							X					X		
rs17740990														
rs1611949														

rs112749152														
rs1667255														
rs1791200	X													
rs1791199														
rs1791198														
rs58114875						X								
rs1667257														
rs77473169														
rs72922962														
rs1667258														
rs11664321														
rs1791197														
rs57844152														
rs117591375														X
rs1791196						X								
rs145583622			X										X	
rs4799585														
rs9951917														
rs12458967														
rs4799586														
rs9947375														
rs4799587														
rs2595361		X												
rs7232056														
rs11663991									X					
rs9797439														
rs56861741													X	
rs9962358														

B: Clumped datasets for the European ancestry. Color refer to variants in the LD block (r^2 > 0.5; different colors represent different LD blocks) and X = presence of a variant in a specific dataset after the LD clumping analysis.

RSID	Colon - Transverse	Colon - Sigmoid	Esophagus - Muscularis	Esophagus - Mucosa	Heart - Atrial Appendage	Heart - Left Ventricle	Liver	Muscle - Skeletal	Nerve - Tibial	Stomach	Small Intestine - Terminal	Adipose - Subcutaneous	Cells - Transformed fibroblasts	Skin - Sun Exposed (Lower leg)
rs8090089	X													
rs4799579														
rs17660933														
rs11081702														
rs1667238														
rs2034404														
rs1667239														
rs1375445														
rs1791191					X	X	X							
rs1375446														

rs1667241					X	X	X							
rs2704058														
rs2704059					X	X	X							
rs9956836														
rs1667242														
rs9956858	X													
rs1791190														
rs75604963														
rs1667243					X	X								
rs11876866	X													
rs1791188					X	X	X							
rs6506932														
rs7243588														
rs1616887														
rs11662311														
rs4799580														
rs16962173	X													
rs77507420														
rs2420813	X													
rs118008746														
rs4799581														
rs8094790														
rs1791187					X	X								
rs1791186														
rs1791185	X													
rs1631020					X	X								
rs79889942														
rs140467514									X		X			
rs16962179	X													
rs12962216														

rs61605601										X				
rs9989534					X	X								
rs149397495	X													
rs113035076			X											
rs2704061					X	X								
rs17740847				X										X
rs73418189					X	X								
rs1574501														
rs60939523														
rs875120														X
rs875119														
rs76431866														
rs1667244														
rs1667245					X		X							
rs76184052														
rs72922938														
rs111395060														
rs73418200										X				
rs80322202												X	X	
rs150555470							X							X
rs3764479														
rs13381522														
rs1667246					X									
rs3764478				X										
rs72922940					X					X			X	
rs3764477														
rs58616646				X										
rs116409170			X											
rs79748512														
rs7231173					X	X								

rs723744														
rs73420308					X	X								X
rs1800458														
rs1080093														
rs72922947														
rs1080094														
rs13381331					X	X								X
rs59882235														
rs75075253														
rs3764476														
rs7235277														
rs3794884														
rs1667250								X						
rs1791227						X								
rs1667251						X								
rs36204272														X
rs62093482											X		X	
rs1791228								X						
rs75032823				X										
rs77973165														
rs113289164														
rs117207032						X								
rs1791229														
rs1667252						X								
rs4799583														
rs1473342														
rs1900880														X
rs1791201														
rs1667253						X								
rs9962355	X													

rs1667254														
rs148278972							X					X		
rs17740990														
rs1611949														
rs112749152														
rs1667255														
rs1791200														
rs1791199														
rs1791198														
rs58114875						X								
rs1667257														
rs77473169														
rs72922962														
rs1667258														
rs11664321														
rs1791197														
rs57844152														
rs117591375														X
rs1791196						X								
rs145583622			X										X	
rs4799585														
rs9951917	X													
rs12458967														
rs4799586														
rs9947375	X													
rs4799587														
rs2595361		X											X	
rs7232056														
rs11663991									X					
rs9797439														

rs56861741													X	
rs9962358														

C: Clumped datasets for the Eastern Asian ancestry. Colors refer to variants in the LD blocks ($r^2 > 0.5$; different colors represent different LD blocks) and X = presence of a variant in a specific dataset after the LD clumping analysis.

**RSID**	**Colon - Transverse**	**Colon - Sigmoid**	**Esophagus - Muscularis**	**Esophagus - Mucosa**	**Heart - Atrial Appendage**	**Heart - Left Ventricle**	**Liver**	**Muscle - Skeletal**	**Nerve - Tibial**	**Stomach**	**Small Intestine - Terminal**	**Adipose - Subcutaneous**	**Cells - Transformed fibroblasts**	**Skin - Sun Exposed (Lower leg)**
rs8090089	X													
rs4799579														
rs17660933														
rs11081702														
rs1667238														
rs2034404														
rs1667239														
rs1375445														
rs1791191														
rs1375446														
rs1667241					X	X								
rs2704058														
rs2704059														
rs9956836														
rs1667242														

rs9956858	X													
rs1791190														
rs75604963														
rs1667243														
rs11876866	X													
rs1791188														
rs6506932														
rs7243588														
rs1616887														
rs11662311														
rs4799580														
rs16962173	X													
rs77507420														
rs2420813	X													
rs118008746														
rs4799581														
rs8094790														
rs1791187														
rs1791186														
rs1791185	X													
rs1631020														
rs79889942														
rs140467514								X		X				
rs16962179	X													
rs12962216														
rs61605601										X				
rs9989534					X	X								
rs149397495	X													
rs113035076			X											
rs2704061														

rs17740847				X										X
rs73418189					X	X								
rs1574501														
rs60939523														
rs875120														X
rs875119														
rs76431866														
rs1667244														
rs1667245							X							
rs76184052														
rs72922938														
rs111395060														
rs73418200										X				
rs80322202												X	X	
rs150555470							X							X
rs3764479														
rs13381522														
rs1667246														
rs3764478														
rs72922940					X					X			X	
rs3764477														
rs58616646				X										
rs116409170			X											
rs79748512														
rs7231173					X	X								
rs723744														
rs73420308					X	X								X
rs1800458														
rs1080093														
rs72922947														

rs1080094														
rs13381331					X	X								X
rs59882235														
rs75075253														
rs3764476														
rs7235277														
rs3794884														
rs1667250								X						
rs1791227														
rs1667251														
rs36204272														X
rs62093482								X			X		X	
rs1791228								X						
rs75032823														
rs77973165														
rs113289164														
rs117207032						X								
rs1791229														
rs1667252														
rs4799583														
rs1473342														
rs1900880														
rs1791201														
rs1667253														
rs9962355	X													
rs1667254														
rs148278972							X					X		
rs17740990														
rs1611949														
rs112749152														

rs1667255														
rs1791200														
rs1791199														
rs1791198														
rs58114875														
rs1667257														
rs77473169														
rs72922962														
rs1667258														
rs11664321														
rs1791197														
rs57844152														
rs117591375														X
rs1791196						X								
rs145583622			X										X	
rs4799585														
rs9951917	X													
rs12458967														
rs4799586														
rs9947375	X													
rs4799587														
rs2595361		X											X	
rs7232056														
rs11663991									X					
rs9797439														
rs56861741													X	
rs9962358														

D: Clumped datasets for the Southern Asian ancestry. Colors refer to variants in the LD blocks (r^2 > 0.5; different colors represent different LD blocks) and X = presence of a variant in a specific dataset after the LD clumping analysis.

RSID	Colon - Transverse	Colon - Sigmoid	Esophagus - Muscularis	Esophagus - Mucosa	Heart - Atrial Appendage	Heart - Left Ventricle	Liver	Muscle - Skeletal	Nerve - Tibial	Stomach	Small Intestine - Terminal	Adipose - Subcutaneous	Cells - Transformed fibroblasts	Skin - Sun Exposed (Lower leg)
rs8090089	X													
rs4799579														
rs17660933														
rs11081702														
rs1667238														
rs2034404														
rs1667239														
rs1375445														
rs1791191														
rs1375446														
rs1667241					X	X								
rs2704058														
rs2704059														
rs9956836														
rs1667242														

rs9956858	X													
rs1791190														
rs75604963														
rs1667243														
rs11876866	X													
rs1791188														
rs6506932														
rs7243588														
rs1616887														
rs11662311														
rs4799580														
rs16962173	X													
rs77507420														
rs2420813	X													
rs118008746														
rs4799581														
rs8094790														
rs1791187														
rs1791186														
rs1791185	X													
rs1631020														
rs79889942														
rs140467514									X		X			
rs16962179	X													
rs12962216														
rs61605601											X			
rs9989534					X	X								
rs149397495	X													
rs113035076			X											
rs2704061														

rs17740847				X										X
rs73418189					X	X								
rs1574501														
rs60939523														
rs875120														X
rs875119														
rs76431866														
rs1667244														
rs1667245							X							
rs76184052														
rs72922938														
rs111395060														
rs73418200										X				
rs80322202												X	X	
rs150555470							X							X
rs3764479														
rs13381522														
rs1667246														
rs3764478														
rs72922940					X					X			X	
rs3764477														
rs58616646				X										
rs116409170			X											
rs79748512														
rs7231173					X	X								
rs723744	X													
rs73420308					X	X								X
rs1800458														
rs1080093														
rs72922947														

rs1080094														
rs13381331					X	X								X
rs59882235														
rs75075253														
rs3764476														
rs7235277														
rs3794884														
rs1667250								X						
rs1791227														
rs1667251														
rs36204272														X
rs62093482								X			X		X	
rs1791228								X						
rs75032823														
rs77973165														
rs113289164														
rs117207032						X								
rs1791229														
rs1667252														
rs4799583														
rs1473342														
rs1900880														
rs1791201														
rs1667253														
rs9962355	X													
rs1667254														
rs148278972							X					X		
rs17740990														
rs1611949														
rs112749152														

rs1667255														
rs1791200														
rs1791199														
rs1791198														
rs58114875														
rs1667257														
rs77473169														
rs72922962														
rs1667258														
rs11664321														
rs1791197														
rs57844152														
rs117591375														X
rs1791196														
rs145583622			X										X	
rs4799585														
rs9951917	X													
rs12458967														
rs4799586														
rs9947375	X													
rs4799587														
rs2595361		X												
rs7232056														
rs11663991									X					
rs9797439														
rs56861741													X	
rs9962358														

E: Clumped datasets for the American ancestry. Colors refer to variants in the LD blocks (r^2 > 0.5; different colors represent different LD blocks) and X = presence of a variant in a specific dataset after the LD clumping analysis.

RSID	Colon - Transverse	Colon - Sigmoid	Esophagus - Muscularis	Esophagus - Mucosa	Heart - Atrial Appendage	Heart - Left Ventricle	Liver	Muscle - Skeletal	Nerve - Tibial	Stomach	Small Intestine - Terminal	Adipose - Subcutaneous	Cells - Transformed fibroblasts	Skin - Sun Exposed (Lower leg)

rs8090089														
rs4799579														
rs17660933														
rs11081702														
rs1667238														
rs2034404														
rs1667239														
rs1375445														
rs1791191														
rs1375446														
rs1667241					X	X								
rs2704058														
rs2704059														
rs9956836														
rs1667242														
rs9956858														
rs1791190														
rs75604963														
rs1667243														
rs11876866														
rs1791188														
rs6506932														
rs7243588														
rs1616887														
rs11662311														
rs4799580														
rs16962173														
rs77507420														
rs2420813	X													
rs118008746														

rs4799581														
rs8094790														
rs1791187														
rs1791186														
rs1791185	X													
rs1631020														
rs79889942														
rs140467514									X		X			
rs16962179														
rs12962216														
rs61605601											X			
rs9989534						X								
rs149397495	X													
rs113035076			X											
rs2704061														
rs17740847				X										X
rs73418189														
rs1574501														
rs60939523														
rs875120														X
rs875119														
rs76431866														
rs1667244														
rs1667245							X							
rs76184052														
rs72922938														
rs111395060														
rs73418200										X				
rs80322202												X	X	
rs150555470							X							X

rs3764479														
rs13381522														
rs1667246														
rs3764478														
rs72922940					X					X			X	
rs3764477														
rs58616646				X										
rs116409170			X											
rs79748512														
rs7231173														
rs723744														
rs73420308														
rs1800458														
rs1080093														
rs72922947														
rs1080094														
rs13381331					X									X
rs59882235														
rs75075253														
rs3764476														
rs7235277														
rs3794884														
rs1667250								X						
rs1791227														
rs1667251														
rs36204272														
rs62093482								X			X		X	
rs1791228								X						
rs75032823														
rs77973165														

rs113289164														
rs117207032						X								
rs1791229														
rs1667252														
rs4799583														
rs1473342														
rs1900880														X
rs1791201														
rs1667253														
rs9962355														
rs1667254														
rs148278972							X					X		
rs17740990														
rs1611949														
rs112749152														
rs1667255														
rs1791200														
rs1791199														
rs1791198														
rs58114875														
rs1667257														
rs77473169														
rs72922962														
rs1667258														
rs11664321														
rs1791197														
rs57844152														
rs117591375														X
rs1791196						X								
rs145583622			X										X	

rs4799585														
rs9951917														
rs12458967														
rs4799586														
rs9947375	X													
rs4799587														
rs2595361		X											X	
rs7232056														
rs11663991									X					
rs9797439														
rs56861741													X	
rs9962358													X	
